# ABRO1 suppresses tumourigenesis and regulates the DNA damage response by stabilizing p53

**DOI:** 10.1038/ncomms6059

**Published:** 2014-10-06

**Authors:** Jianhong Zhang, Mengmeng Cao, Jiahong Dong, Changyan Li, Wangxiang Xu, Yiqun Zhan, Xiaohui Wang, Miao Yu, Changhui Ge, Zhiqiang Ge, Xiaoming Yang

**Affiliations:** 1 State Key Laboratory of Proteomics, Beijing Proteomics Research Center, Beijing Institute of Radiation Medicine, 27-Taiping Road, Beijing 100850, China; 2 School of Chemical Engineering and Technology, TianJin University, Tianjin 300073, China; 3 Department of Liver and Gallbladder Surgery, The General Hospital of Chinese People’s Liberation Army, Beijing 100853, China

## Abstract

Abraxas brother 1 (ABRO1) has been reported to be a component of the BRISC complex, a multiprotein complex that specifically cleaves ‘Lys-63’-linked ubiquitin. However, current knowledge of the functions of ABRO1 is limited. Here we report that ABRO1 is frequently downregulated in human liver, kidney, breast and thyroid gland tumour tissues. Depletion of ABRO1 in cancer cells reduces p53 levels and enhances clone formation and cellular transformation. Conversely, overexpression of ABRO1 suppresses cell proliferation and tumour formation in a p53-dependent manner. We further show that ABRO1 stabilizes p53 by facilitating the interaction of p53 with USP7. DNA-damage induced accumulation of endogenous ABRO1 as well as translocation of ABRO1 to the nucleus, and the induction of p53 by DNA damage is almost completely attenuated by ABRO1 depletion. Our study shows that ABRO1 is a novel p53 regulator that plays an important role in tumour suppression and the DNA damage response.

## Abstract (web summary)

The scaffold protein Abraxas brother 1 (ABRO1) accumulates in the nucleus after oxidative stress but its role in cellular responses to DNA damage has not been elucidated. Here the authors show that ABRO1 exerts its tumour suppressor activity by regulating p53 stability via USP7 deubiquitinase.

## Graphical abstract

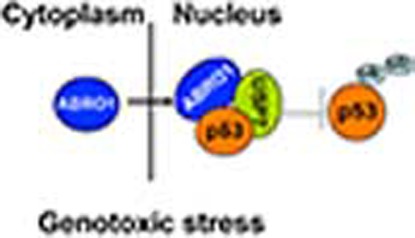

The p53 protein, a product of a tumour suppressor gene, plays a key role in the maintenance of cell homeostasis^1^,^2^,^3^. Under ordinary conditions, p53 is a short-lived protein; its stability is mainly regulated by ubiquitination^4^,^5^,^6^,^7^,^8^,^9^. However, in response to various types of stress, p53 is rapidly stabilized and its downstream target genes are activated to initiate cell cycle arrest, apoptosis, senescence, or differentiation. Thus, p53 mediates the cell’s response to various cellular stressors and plays a pivotal role in tumourigenesis.

The activation, expression and intracellular translocation of p53 are mainly regulated by post-translational modifications such as phosphorylation, acetylation and ubiquitination^10^,^11^,^12^, all of which are known to affect the function of p53. Ubiquitination is a key regulatory event in the p53 pathway and has been the focus of many studies^13^,^14^. Similar to most post-translational modifications, ubiquitination of p53 can be reversed by the counteraction of deubiquitinating enzymes (DUBs)^15^,^16^. USP2a influences cell survival through the regulation of the p53 pathway by stabilizing the activity of MDM2 and MdmX^17^. USP10 is also a key regulator of p53 stability. In unstressed cells, USP10 remains in the cytoplasm, where it directly deubiquitinates p53 and mediates the re-entry of p53 into the nucleus. On DNA damage, USP10 is stabilized and some of it translocates to the nucleus to activate p53. Depletion of USP10 results in increased p53 degradation^18^. USP7, which is also known as herpes-associated ubiquitin-specific protease, has also been shown to stabilize the half-life of p53 by regulating both p53 and its ubiquitin E3 ligase MDM2 (refs 19, 20, 21, 22, 23). Moreover, it has been reported that suppression of USP5 stabilizes p53, whereas it has little or no effect on the stability of MDM2 (ref. 24). Importantly, USP7, USP2a and USP10 contribute to cancer pathogenesis, and therapeutic strategies that target these p53-specific DUBs may become important as cancer treatments^18^,^25^,^26^,^27^,^28^,^29^.

Several proteins have been reported to regulate the deubiquitination of p53 by affecting the interaction between USP7 and MDM2, or that between USP7 and p53. DAXX mediates the stabilizing effect of USP7 on MDM2 by promoting the binding of USP7 and MDM2 (refs 30, 31). RASSF1A has been reported to control the assembly of the USP7/DAXX/MDM2 complex by blocking interactions among MDM2, DAXX and USP7, and by promoting the ubiquitination of MDM2, resulting in stabilization of p53 (ref. 32). TSPYL5 has been shown to physically interact with USP7 and to suppress p53 activity by reducing the activity of USP7 towards p53 (ref. 26). EBNA1 competes with p53 for binding to USP7, consequently reducing the stability of p53 (ref. 33). The retinoic-acid-related orphan nuclear receptor α (*RORα*) is a direct target gene of p53. RORα promotes interactions between p53 and USP7 but does not affect interactions between p53 and MDM2 (ref. 34). The accumulated evidence regarding the interactions of USP7 and MDM2 with p53 indicates that these interactions are important in regulating the stability of p53 under both physiological and pathological conditions. Through its action towards p53, RORα could function as a tumour suppressor^35^,^36^. DAXX plays a critical role in development because of its anti-apoptotic function^37^. In contrast, the expression of TSPYL5 could override the p53-dependent arrest of cell proliferation and oncogene-induced senescence, and contribute to oncogenic transformation^26^. Overexpression of EBNA1 has been found to increase cell survival after DNA damage^38^. There is currently considerable interest in these molecules and their interactions because some of these pathways may represent potential therapeutic targets, and understanding the basis of the interactions between these proteins may help in the design of effective drugs^39^,^40^.

Abraxas brother 1 (ABRO1), also known as KIAA0157 and FAM175B, is present on chromosome 10q26.13. The function of the ABRO1 protein is largely unknown. Recent studies have revealed that ABRO1 is part of the BRISC complex (BRCC36-containing isopeptidase complex), a multi-protein complex that specifically cleaves Lys-63-linked ubiquitin^41^,^42^,^43^,^44^. This complex, which uses ABRO1 instead of Abraxas and does not contain RAP80, appears to represent a major K63-Ub-directed DUB activity in the cytoplasm^41^,^45^. Serine hydroxymethyltransferase has also been shown to be a specific BRISC component that targets the catalytic core of this complex to activate the IFNAR1 chains of the type 1 interferon receptor^46^. ABRO1 knockout mice generated by deleting exons 6 and 7 of the murine ABRO1 locus were shown to be viable and fertile, and to exhibit attenuated responses to interferon^46^. ABRO1 is homologous to CCDC98/Abraxas, sharing 39% sequence homology within the amino-terminal region, which contains a structural domain that interacts with subunits NBA1, BRE and BRCC36 (refs 43, 47, 48, 48). Similar to BRCA1, CCDC98 plays a role in radiation sensitivity and DNA damage-induced G2/M checkpoint control^49^. Although similar to CCDC98, the carboxy-terminal region of ABRO1 lacks the PSXXF domain that serves as a specific recognition motif for the BRCT domain of BRCA1 (ref. 47). For this reason, ABRO1 does not interact with BRCA1.

To date, no study has established whether ABRO1 participates in the cellular response to DNA damage. Recently, ABRO1 has been reported to interact with several transcription factors, including THAP5 (ref. 45), ATF4, ATF5 and JunD proteins^50^. Although ABRO1 is predominantly cytoplasmic, it enters the nucleus during oxidative stress^50^. The ability of ABRO1 to translocate into the cell nucleus following oxidative stress and to specifically interact with a number of transcription factors provides a potential avenue through which its cellular functions might be exploited. In addition, a global proteomic analysis of DUBs and their associated protein complexes has identified USP7 as a candidate binding partner for ABRO1 (ref. 51).

In this paper, we report that ABRO1 controls the DUB activity of USP7 on p53 and demonstrate that ABRO1 plays a role in tumorigenesis and in the DNA damage response. Our data support the notion that ABRO1 is a novel p53 regulator that plays an important role in tumour suppression and in the DNA damage response.

## Results

### ABRO1 is downregulated in several human cancer tissues

**Figure 1:**
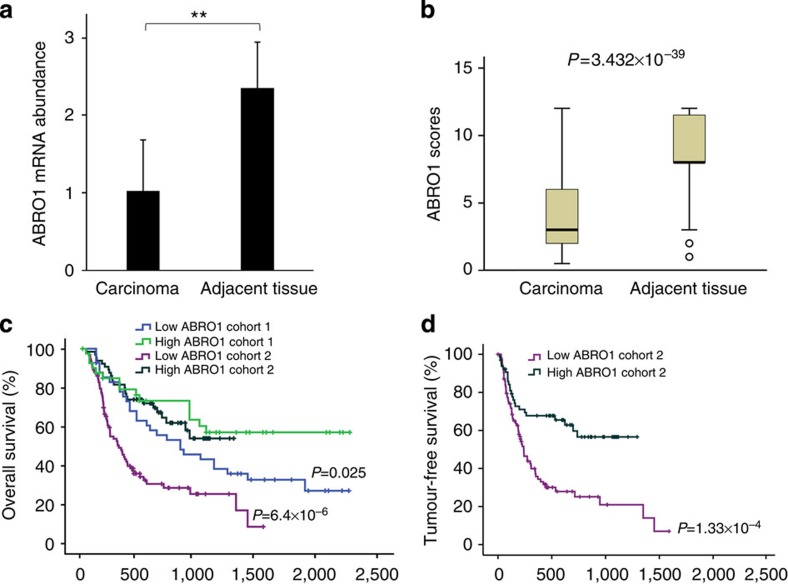
ABRO1 is frequently downregulated in human liver cancer tissues. (**a**) Quantitative RT–PCR analysis of ABRO1 mRNA levels in liver cancer tissues and corresponding adjacent tissue from 22 HCC patients. Data were analysed using Student’s *t-*test and are shown as the mean±s.d. **P<0.01. (**b**) ABRO1 expression scores shown as box plots, with the horizontal lines representing the median; the bottoms and tops of the boxes represent the 25th and 75th percentiles, respectively, and the vertical bars represent the range of data. Comparison was performed using the Mann–Whitney *U*-test. *n*=310. (**c**,**d**) Low ABRO1 expression correlates with poor survival of HCC patients. Kaplan–Meier survival curves of overall survival in Cohort 1 and Cohort 2 (**c**) and tumour-free survival in Cohort 2 (**d**) are shown. Comparison was made between groups with high ABRO1 expression (score≥9) and low ABRO1 expression (score<9). The marks on the graph lines represent censored samples. *P*-value refers to two-sided log-rank tests.

To investigate the role of ABRO1 in human cancers, we evaluated the expression of ABRO1 in several tumours and in their corresponding normal tissues. We found that ABRO1 messenger RNA was significantly downregulated in tumour samples derived from the thyroid gland (eight of ten), liver (three of three), kidney (five of ten) and breast (five of ten) (Supplementary Fig. 1a). The downregulation of ABRO1 mRNA in hepatocellular carcinoma (HCC) tissues was confirmed by quantitative reverse transcriptase–PCR (qRT–PCR) in 22 pairs of HCC specimens and in the adjacent non-cancerous liver tissues (Fig. 1a and Supplementary Fig. 1b). To elucidate the clinical relevance of ABRO1 downregulation, we analysed the expression levels of ABRO1 in a cohort of 310 hepatic cancer specimens using immunohistochemical analysis with an *ABRO1*-specific antibody. The antibody specificity was verified (Supplementary Fig. 1c). Representative staining is shown in Supplementary Fig. 1d. We found that ABRO1 expression was significantly downregulated in hepatic tumour tissues relative to matched adjacent normal tissues (Fig. 1b, *P*=3.432 × 10^−39^). Similar results were also obtained for thyroid gland, breast and renal cell cancer tissues compared with their respective adjacent normal tissues (Supplementary Fig. 2 and Table 1).

To further investigate whether downregulation of ABRO1 is correlated with survival in HCC patients, two independent cohorts of HCC patients, Cohort 1 (90 patients) and Cohort 2 (161 patients), were analysed. The HCC characteristics of these patients are listed in Supplementary Data 1. Interestingly, survival analysis indicated that low ABRO1 levels in HCC tissues (score<9) significantly correlated with shorter survival time of the patients after surgery and treatment (Fig. 1c,d). We also performed Cox proportional hazards regression analysis to exclude the confounder effect. Multivariate analysis confirmed that low ABRO1 expression was an independent predictor for reduced tumour-free survival of HCC patients (Supplementary Tables 1,2). Taken together, these clinical data indicate that downregulation of ABRO1 is a critical event in tumour progression and that ABRO1 may be a single prognostic marker for disease outcome in HCC.

### ABRO1 suppresses tumourigenesis in a p53-dependent manner

**Figure 2:**
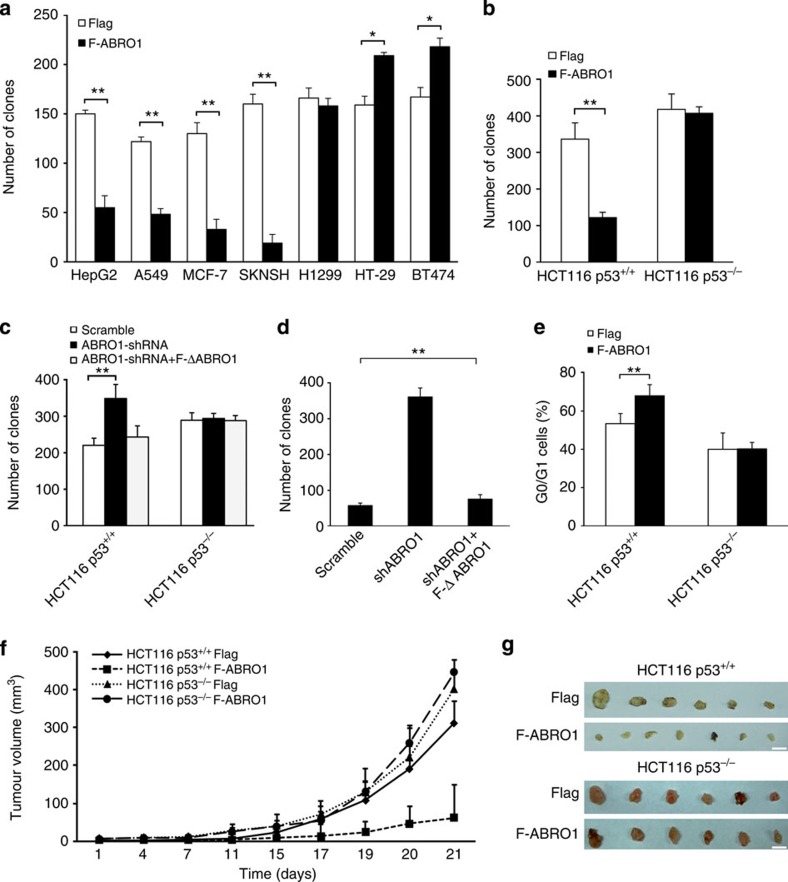
ABRO1 suppresses cell growth in a p53-dependent manner. (**a**) Cells were transfected with pCMV-Flag-ABRO1 or pCMV-Flag vectors, and 2 × 10^3^ cells were plated per 6-cm dish and selected with G418. Clone numbers (>50 cells) were determined after 2 weeks. The data shown are the means±s.d. of three independent experiments and were analysed using Student’s *t-*test. *P<0.05, **P<0.01. (**b**) HCT116 p53^+/+^ and HCT116 p53^−/−^ cells were transfected with pCMV-Flag-ABRO1 or pCMV-Flag vectors, and the number of G418-resistant clones was determined. The data shown are the means±s.d. of three independent experiments and were analysed using Student’s *t-*test. **P<0.01. A representative image of the clones is shown in Supplementary Fig. 3a. (**c**) HCT116 p53^+/+^ and HCT116p53^−/−^ cells were infected with control lentivirus, with lentivirus encoding shRNA specific to ABRO1 or with lentivirus encoding ABRO1 shRNA and RNAi-immune ABRO1. Clones were counted 2 weeks after initial plating. The data shown are the means±s.d. of three independent experiments and were analysed using Student’s *t-*test. **P<0.01. A representative image of the clones is shown in Supplementary Fig. 3b. (**d**) L02 cells were infected with control lentivirus, with lentivirus encoding shRNA specific to ABRO1 or with lentivirus encoding ABRO1 shRNA and RNAi-immune ABRO1, and plated on soft agar. Clones were counted 2 weeks after initial plating. The data shown are the means±s.d. of three independent experiments and were analysed using Student’s *t-*test. **P<0.01. A representative image of the clones is shown in Supplementary Fig. 3c. (**e**) HCT116 p53^+/+^ and HCT116 p53^−/−^ cells were transfected with pCMV-Flag-ABRO1 or pCMV-Flag vectors, and G418-resistant cells were selected. The cells were pulsed for 1 h with BrdU before being harvested, fixed, stained with anti-BrdU fluorescein isothiocyanate (FITC) and propidium iodide (PI), sorted by flow cytometry and analysed for DNA content. The data shown are the mean±s.d. of three independent experiments and were analysed using Student’s *t-*test. **P<0.01. (**f**) Volume of xenograft tumours derived from control or ABRO1-overexpressing HCT116 cells. *n*=10 in each group. Tumour sizes were measured using a calliper at the indicated time points. The data shown are means±s.d. (**g**) Excised tumours from control or ABRO1-overexpressing HCT116 cells. Scale bar, 10 mm.

The fact that ABRO1 protein levels were markedly decreased in a subset of primary human cancers suggests that ABRO1 may have a role in the prevention of tumour formation. To determine the effects of ABRO1 on tumour cell growth, an ABRO1 expression vector was transfected into cells of the human HCC cell line HepG2, the human breast cancer cell lines MCF-7 and BT474, the human neuroblastoma cell line SKNSH, the human non-small cell lung cancer cell line A549, the human lung cancer cell line H1299 and the human colon cancer cell line HT-29, and G418-resistant clones were established. The results indicated that enforced expression of ABRO1 significantly inhibited the formation of clones by HepG2, MCF7, A549 and SKNSH cells. However, in H1299 cells, in which p53 is completely absent^52^, enforced expression of ABRO1 did not significantly affect clone formation (Fig. 2a). Moreover, increased expression of ABRO1 in HT-29 and BT474 cells, which contain mutant p53, resulted in increased clone formation (Fig. 2a). These results indicate that ABRO1 may be a growth suppressor gene that is functionally related to p53.

To determine whether ABRO1 inhibits cell growth in a p53-dependent manner, a vector expressing ABRO1 was transfected into HCT116 p53^+/+^ and HCT116 p53^−/−^ cells, and the number of G418-resistant clones was determined. The results revealed that overexpression of ABRO1 led to a decrease of 70% in clone formation by HCT116 p53^+/+^ cells. In contrast, overexpression of ABRO1 had little effect on clone formation by HCT116 p53^−/−^ cells (Fig. 2b and Supplementary Fig. 3a). Conversely, depletion of ABRO1 using lentiviral delivery of short hairpin RNA (shRNA) led to the formation of larger numbers of clones with HCT116 p53^+/+^cells but not with HCT116 p53^−/−^ cells (Fig. 2c and Supplementary Fig. 3b). Expression of a mutant ABRO1 that is resistant to *ABRO1*-specific RNA interference (RNAi) abrogated the effect of RNAi on cell clone formation, suggesting that RNAi inhibition of cell clone formation was not due to off-target effects of RNAi (Fig. 2c and Supplementary Fig. 3b). To determine whether endogenous ABRO1 protects against cellular transformation, anchorage-independent growth was measured after inhibition of ABRO1 expression by lentivirus-delivered shRNA in immortalized but not transformed L02 cells, a cell line that originated from human normal liver cells. As shown in Fig. 2d and Supplementary Fig. 3c, knockdown of ABRO1 resulted in an approximately sixfold increase in clone number and in the production of larger clones compared with cells infected with control lentivirus. This increase in clone number was completely attenuated by RNAi-immune ABRO1. Downregulation of ABRO1 was confirmed in a portion of the cells before plating in soft agar (Supplementary Fig. 3c).

To determine the effects of ABRO1 on the cell cycle, HCT116 p53^+/+^ and HCT116 p53^−/−^ cells were transfected with pCMV-Flag-ABRO1 or pCMV-Flag vectors and the G418-resistant cells were selected and analysed by simultaneous flow cytometric analysis for DNA synthesis (5-bromodeoxyuridine (BrdU) incorporation) and DNA content (propidium iodide staining). As shown in Fig. 2e and Supplementary Fig. 3d, overexpression of ABRO1 significantly increased G0/G1 arrest in HCT116 p53^+/+^ cells but not in HCT116 p53^−/−^ cells. To investigate whether ABRO1 regulates tumourigenesis *in vivo*, we injected the transfected cells into nude mice and observed tumour growth. Ectopic ABRO1 expression in HCT116 p53^+/+^ cells, but not in p53^−/−^ cells, significantly suppressed tumour progression in immune-deficient mice (Fig. 2f,g). Importantly, western blotting of excised tumours revealed that ABRO1, p53, p21, puma and cleaved caspase 3 were elevated in tumours originating from HCT116 p53^+/+^ cells (Supplementary Fig. 3e). Taken together, these data suggest that ABRO1 suppresses tumour cell growth in a p53-dependent manner and that it might represent a candidate tumour suppressor.

### ABRO1 increases apoptosis via p53 in response to DNA damage

**Figure 3:**
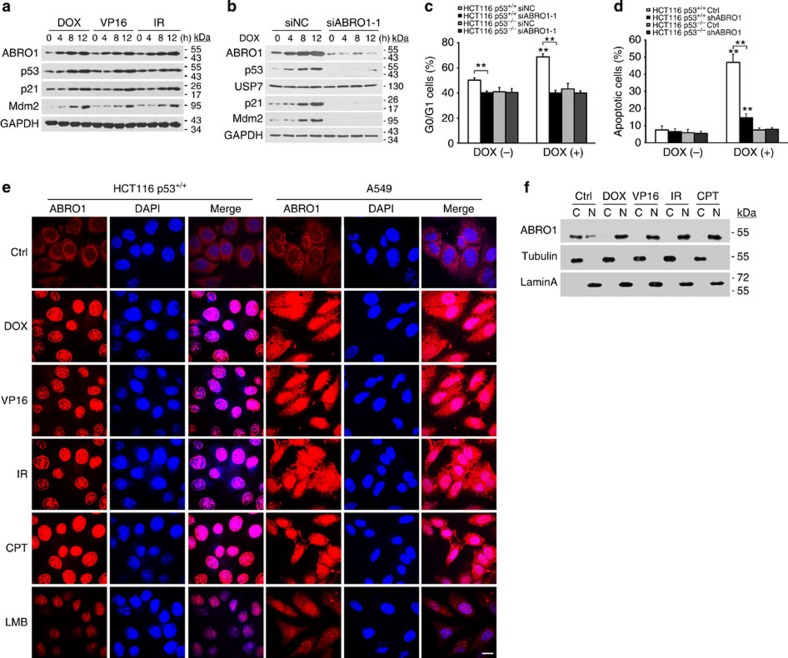
ABRO1 translocates to the nucleus after DNA damage and regulates the p53-dependent DNA damage response. (**a**) HCT116 p53^+/+^ cells were treated with DOX (2 μg ml^−1^), VP16 (20 μM), or irradiation (10 Gy) for the indicated times. Proteins were extracted and subjected to western blotting. (**b**) HCT116 p53^+/+^ cells were transfected with siABRO1-1 or siNC. After 48 h, cells were treated with DOX (2 μg ml^−1^) for the indicated times. Proteins were extracted and subjected to western blotting. (**c**) HCT116 cells were transfected with siABRO1-1 or siNC. Forty-eight hours after transfection, the cells were treated with DOX (2 μg ml^−1^) for 12 h, and cell cycle profiles were determined by FACS as described in the legend to Fig. 2. The data shown are the means±s.d. of three independent experiments and were analysed using Student’s *t-*test. **P<0.01. (**d**) HCT116 cells were infected with control lentivirus or with lentivirus encoding shRNA specific to ABRO1 and treated with DOX (2 μg ml^−1^) for 12 h. Cell apoptosis was examined by FACS. The values shown are the mean±s.d. of three independent experiments; data were analysed using Student’s *t-*test. **P<0.01. (**e**) HCT116 p53^+/+^ and A549 cells were treated with DOX (2 μg ml^−1^), VP16 (20 μM), IR (10 Gy) or CPT (2 μM). The subcellular localization of ABRO1 was detected by immunochemistry with ABRO1 antibody, and 4',6-diamidino-2-phenylindole (DAPI) was used to stain the nucleus. Leptomycin B (LMB) was used as a positive control. Scale bar, 10 μm. (**f**) HCT116 p53^+/+^ cells were treated as indicated. After harvesting and fractionation of the cells, cellular fractions were blotted with the indicated antibodies. (C, cytoplasmic; N, nuclear).

To provide further insight into the biological function of ABRO1, we identified the upstream signals responsible for induction of ABRO1. HCT116 p53^+/+^ cells were treated with DNA-damaging agents such as doxorubicin (DOX), etoposide (VP16) and ionizing radiation (IR), and examined to determine whether these agents induce ABRO1 expression. Intriguingly, DOX, VP16 and IR exposure increased the expression of ABRO1 as well as that of p53, p21 and MDM2, gene products that are known to be induced by DNA-damaging agents (Fig. 3a). In contrast, upregulated expression of p53, p21 and MDM2 by DOX treatment was not observed after transfection of the cells with ABRO1 small interfering RNA (siRNA) (Fig. 3b). These results indicate that ABRO1 may participate in the upregulation of p53 induced by DNA damage.

We also examined whether ABRO1 induction by DNA-damaging agents depends on p53 activation. Induction of ABRO1 also occurred in HCT116 p53^−/−^ cells after DOX treatment, indicating that ABRO1 induction is p53-independent (Supplementary Fig. 4a). Furthermore, we investigated whether ABRO1 regulates DNA damage-mediated cell cycle arrest and apoptosis. Flow cytometric analysis revealed that knockdown of ABRO1 in HCT116 p53^+/+^ cells abrogated the DOX-induced increase of sub-G1 phase cells and attenuated p53-mediated apoptosis. However, in HCT116 p53^−/−^ cells, knockdown of ABRO1 failed to alter DOX-induced sub-G1 phase cells or apoptosis (Fig. 3c,d and Supplementary Fig. 4b,c). These results are consistent with the observed decreased expression of p53 and p21 in cells with ABRO1 downregulation (Fig. 3b) and support the idea that p53 cooperates with ABRO1 in mediating apoptosis.

Under normal conditions, ABRO1 is primarily located in the cytoplasm, with some of the protein found in the nucleus (Fig. 3e). As most DNA damage signalling is thought to occur in the nucleus, we investigated whether ABRO1 translocates into the nucleus following DNA damage. We treated HCT116 p53^+/+^ and A549 cells with DOX, VP16, IR or camptothecin (CPT) and examined the subcellular localization of ABRO1 by immunocytochemistry. As shown in Fig. 3e, ABRO1 translocated into the nucleus after cells were treated with DNA-damaging agents. Nuclear translocation of ABRO1 after DNA damage was confirmed by cell fractionation assays (Fig. 3f). Leptomycin B treatment also induced ABRO1 nuclear localization, suggesting that ABRO1 is actively exported out of the nucleus in unstressed cells. These results indicate that significant nuclear translocation of ABRO1 occurs at the time of DNA damage.

### ABRO1 increases the stability of p53 protein

**Figure 4:**
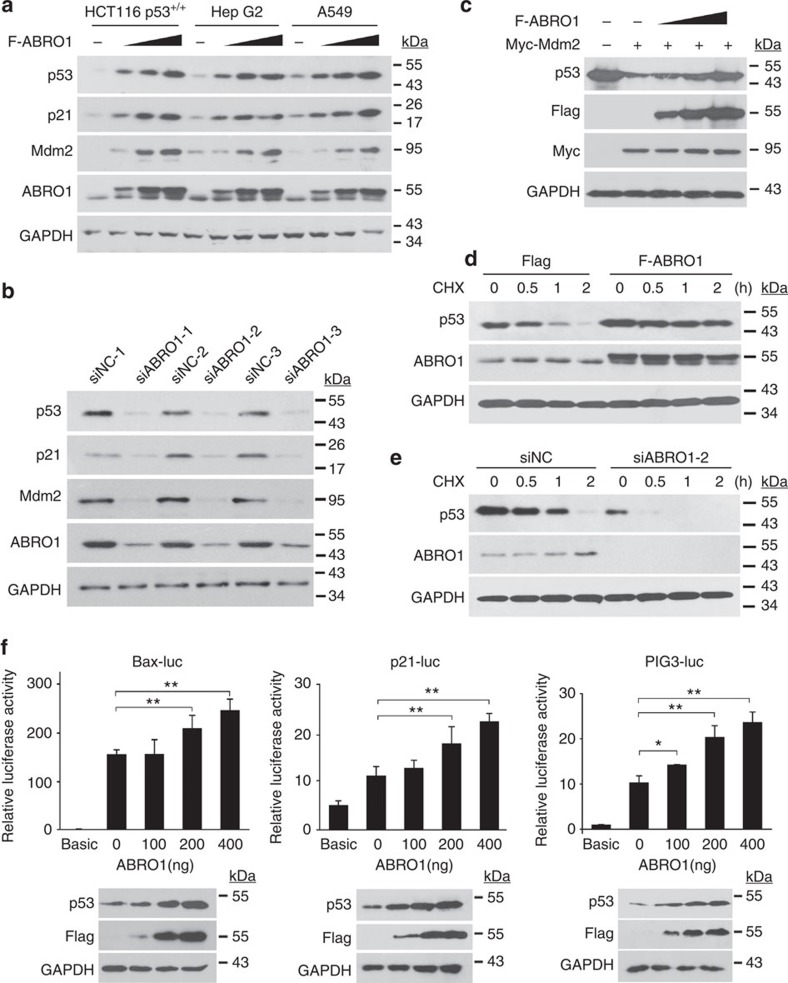
ABRO1 increases the protein stability of p53. (**a**). HCT116 p53^+/+^, HepG2 and A549 cells were transfected with increasing concentrations of plasmid encoding Flag–ABRO1. After 36 h, proteins were extracted and subjected to western blotting. (**b**) HCT116 p53^+/+^ cells were transfected with different ABRO1 siRNAs and siNC. After 36 h, proteins were extracted and subjected to western blotting. (**c**) HCT116 p53^+/+^ cells were co-transfected with plasmids encoding Flag-ABRO1 and Myc-MDM2. Proteins were extracted and subjected to western blotting. (**d**) HCT116 p53^+/+^ cells were treated with cycloheximide (CHX) (1 μg μl^−1^) at the indicated times after transfection with pCMVFlag-ABRO1. Proteins were extracted and subjected to western blotting. (**e**) HCT116 p53^+/+^ cells were treated with CHX (1 μg μl^−1^) at the indicated times after transfection with siABRO1-2 or siNC. Proteins were extracted and subjected to western blotting. (**f**) HCT116 p53^+/+^ cells were co-transfected with the indicated constructs, and reporter activity was determined as described in Experimental Procedures. Data were generated from three independent experiments, each performed in triplicate (The data shown are the mean±s.d.). Data were analysed using Student’s *t-*test.*P<0.05, **P<0.01.

As ABRO1 affects cell functions in a p53-dependent manner, we investigated whether it affects the stability or transcriptional activity of p53. As shown in Fig. 4a, enforced expression of ABRO1 led to the accumulation of endogenous p53 protein in HCT 116 p53^+/+^, HepG2 and A549 cells in a dose-dependent manner. Consistent with this observation, ABRO1 overexpression also led to increased expression of MDM2 and p21, downstream targets of p53. The level of p53 protein was also dramatically increased when ABRO1 was co-transfected with the p53 expression vector in HCT116 p53^−/−^ cells (Supplementary Fig. 5a). This effect of ABRO1 on p53 level was not due to changes in transcription, because ABRO1 did not alter the abundance of p53 mRNA in these cells, whereas the mRNA levels of p21 and MDM2 changed markedly (Supplementary Fig. 5b). Conversely, depletion of ABRO1 by transfection of ABRO1 siRNAs resulted in significant downregulation of expression of p53, p21 and MDM2 (Fig. 4b). Degradation of p53 by the E3 ubiquitin ligase MDM2 is the major cellular mechanism for regulation of p53 (ref. 6). As shown in Fig. 4c, overexpression of MDM2 indeed induced significant degradation of p53; however, co-expression of ABRO1 effectively rescued p53 from MDM2-induced degradation.

To determine whether ABRO1 affects p53 stability, control cells and cells overexpressing ABRO1 were treated with the protein synthesis inhibitor CHX for various times. As Fig. 4d shows, the p53 protein level in control cells decreased by ~90% within 2 h after CHX treatment. However, in cells overexpressing ABRO1, only a small decrease in p53 protein level was observed. Consistently, the half-life of the p53 protein was reduced by depletion of endogenous ABRO1 using ABRO1 siRNA after treatment with CHX (Fig. 4e).

To further explore the role of ABRO1 in the regulation of p53, reporter assays were performed following expression of ABRO1. As shown in Fig. 4f, ectopic expression of ABRO1 in HCT116 p53^+/+^ cells increased p53-mediated transcription activity in a dose-dependent manner as measured with a PIG3 p53 response reporter. Similar results were obtained with p53-responsive luciferase reporters such as Bax and p21 promoter-driven luciferase constructs. Taken together, these results indicate that ABRO1 regulates p53 protein stability and increases the transcriptional activity of p53.

**Figure 5:**
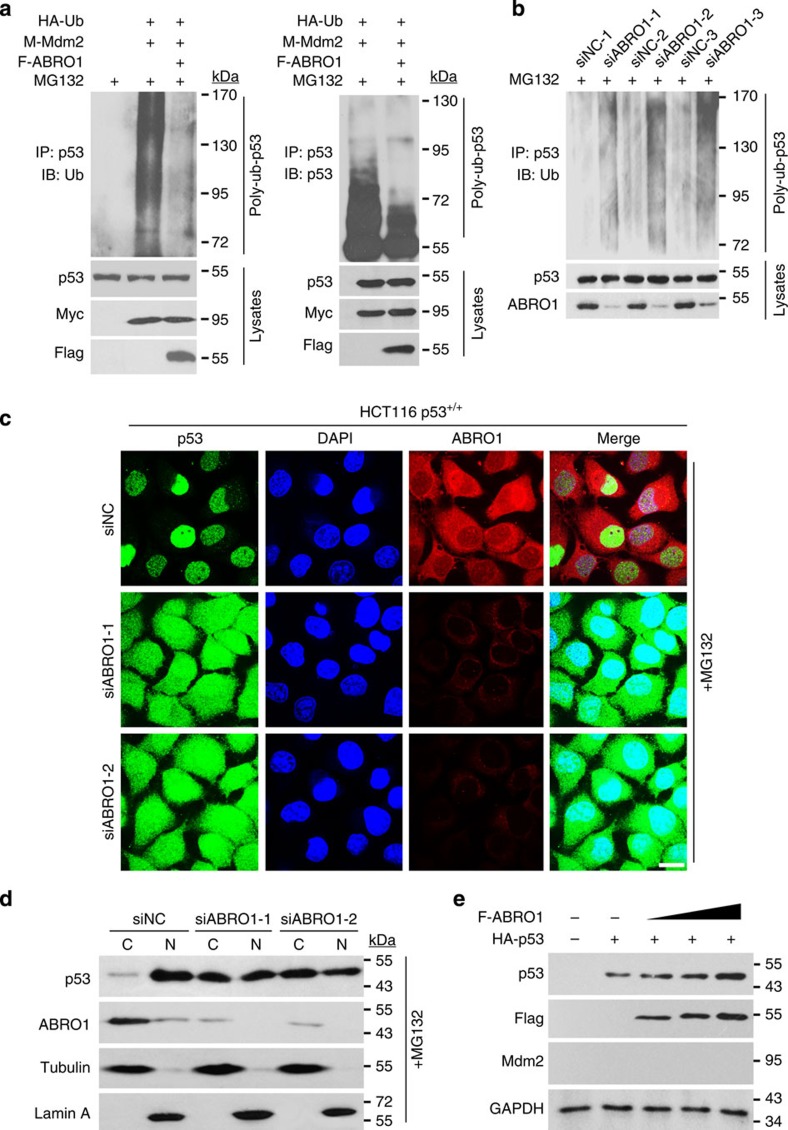
ABRO1 regulates p53 stability by inhibiting p53 ubiquitination. (**a**) HCT 116 p53^+/+^ cells were co-transfected with the indicated constructs. Twenty-four hours later, the cells were treated with 20 μM MG-132 for 4 h. Proteins were extracted, immunoprecipitated with p53 (FL-393) antibody and immunoblotted with anti-Ub antibody (left) or p53 antibody (DO-1) (right). (**b**) HCT 116 p53^+/+^ cells were transfected with various ABRO1 siRNAs or with siNC. After 48 h, the cells were treated with 20 μM MG-132 for 4 h before harvesting and lysis for immunoprecipitation with p53 (FL-393) antibody and immunoblotting with anti-Ub antibody. (**c**) HCT 116 p53^+/+^ cells were seeded onto small slide glass coverslips in 24-well culture plates and transfected with ABRO1 siRNAs or siNC. Forty-eight hours later, the cells were treated with 20 μM MG-132 for 4 h and the subcellular localization of p53 was detected by immunochemistry with p53 antibody. Scale bar, 10 μm. (**d**) HCT 116 p53^+/+^ cells were treated as in **c**. The nuclear protein fractionation assay was performed, and the samples were immunoblotted with the indicated antibodies. (**e**) MEF mdm2^−/−^/p53^−/−^ cells were co-transfected with Flag-ABRO1 and HA-p53 plasmids. Proteins were extracted and subjected to western blotting.

As p53 is degraded through the ubiquitin–proteasome pathway^6^, we next examined whether ABRO1 affects p53 ubiquitination. As shown in Fig. 5a, co-expression of ubiquitin and MDM2 significantly triggered p53 ubiquitination. However, p53 ubiquitination was diminished by co-expression of ABRO1. To determine whether ABRO1 affects p53 ubiquitination at the endogenous level, HCT116 p53^+/+^ cells were transfected with ABRO1 siRNAs followed by treatment with MG132. As shown in Fig. 5b, knockdown of ABRO1 dramatically increased p53 ubiquitination, indicating that ABRO1 inhibits p53 ubiquitination.

Previous studies have suggested that ubiquitination of p53 by MDM2 could induce the migration of p53 from the nucleus to the cytoplasm^18^. We therefore evaluated whether ABRO1 influences the subcellular localization of p53 after enforced MDM2 expression. In control cells, p53 was readily detected in the nucleus. In cells transfected with MDM2, we observed nuclear export of endogenous p53, and this export could be blocked by Leptomycin B treatment. However, co-expression of ABRO1 with MDM2 reversed the MDM2-induced cytoplasmic translocation of p53 (Supplementary Fig. 6a). This result was confirmed using cell fractionation assays (Supplementary Fig. 6b). Furthermore, the results of both immunochemistry and cell fractionation assays showed that ABRO1 knockdown via siRNAs caused p53 cytoplasmic translocation (Fig. 5c,d). These results suggest that ABRO1 counteracts the action of MDM2 and induces p53 translocation from the cytoplasm back to the nucleus.

### ABRO1 regulates p53 stability independently of MDM2

As MDM2 ubiquitin E3 ligase has been shown to trigger p53 ubiquitination and degradation, we examined whether ABRO1 inhibits p53 ubiquitination and stabilizes p53 by modulating MDM2 function. We first measured the level of MDM2 protein in cells overexpressing ABRO1. As shown in Supplementary Fig. 6c, the overexpression of ABRO1 in HCT116 p53^+/+^ cells increased the protein level of MDM2, whereas changes in the MDM2 protein level was not observed in HCT116 p53^−/−^ cells. Furthermore, we found that overexpression of ABRO1 in HCT116 p53^+/+^ cells significantly increased the MDM2 mRNA level (Supplementary Fig. 5b), indicating that the increased MDM2 protein level might be regulated by p53 transcription. Neither the ectopic expression of ABRO1 nor the knockdown of ABRO1 was found to affect the stability of MDM2 protein (Supplementary Fig. 6d,e). The co-immunoprecipitation (Co-IP) assay also showed no interaction between ABRO1 and MDM2, and overexpression of ABRO1 failed to affect the mutual binding of p53 and MDM2 (Supplementary Fig. 6f). Consistent with these data, p53 was found to be upregulated in mouse embryonic fibroblasts (MEF) deficient in both p53 and Mdm2 (*p53*^*–/–*^*Mdm2*^*–/–*^) when ABRO1 was co-expressed with p53 (Fig. 5e). These results suggested that ABRO1 directly stabilizes p53 independently of MDM2.

### ABRO1 forms a complex with USP7 and p53

**Figure 6:**
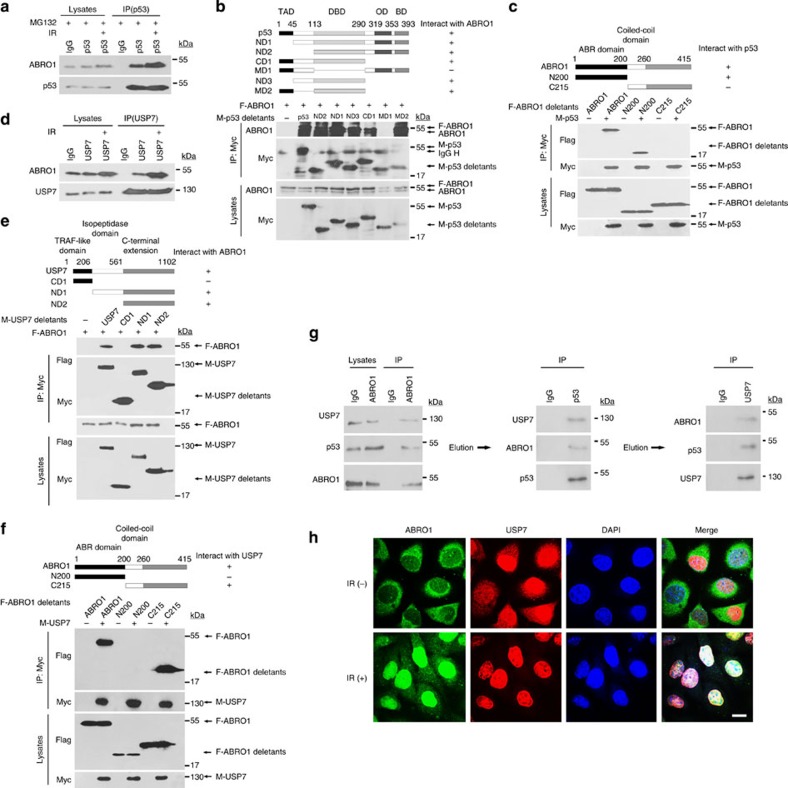
ABRO1 forms a complex with USP7 and p53. (**a**) Lysates of HCT116 p53^+/+^ cells with or without exposure to IR (10 Gy) were subjected to immunoprecipitation with control IgG or anti-p53 antibody. The immunoprecipitates were then blotted with ABRO1 antibody. (**b**) 293 cells were co-transfected with the indicated constructs, and lysates were subjected to immunoprecipitation with anti-myc. The immunoprecipitates were then blotted with ABRO1 antibody and anti-myc antibody. (**c**) 293 cells were co-transfected with the indicated constructs, and lysates of the cells were subjected to immunoprecipitation with anti-myc. The immunoprecipitates were then blotted with anti-Flag antibody and anti-myc antibody. (**d**) Lysates of HCT116 p53^+/+^ cells with or without exposure to IR (10 Gy) were subjected to immunoprecipitation with control IgG and anti-USP7. The immunoprecipitates were then blotted with ABRO1 antibody. (**e**,**f**) 293 cells were co-transfected with the indicated constructs, and cell lysates were subjected to immunoprecipitation with anti-myc. The immunoprecipitates were then blotted with anti-Flag antibody and anti-myc antibody. (**g**) HCT116 p53^+/+^ cell lysates were subjected to immunoprecipitation with control IgG or anti-ABRO1 antibody. The immunoprecipitates were washed with high-salt elution buffer and sequential immunoprecipitation was performed with the indicated antibodies. (**h**) HCT 116 p53^+/+^ cells were seeded on glass coverslips in 24-well cell culture plates and then treated with or without IR. The subcellular localization of ABRO1, USP7 and p53 was detected by immunochemistry using the indicated antibodies. Scale bars, 10 μm.

As ABRO1 regulates p53 stability, we investigated whether ABRO1 interacts with p53. Interaction between endogenous ABRO1 and endogenous p53, examined by Co-IP assay, was observed in HCT116 p53^+/+^ cells (Fig. 6a) but not in HCT116 p53^−/−^ cells. Consistent with the results showing that DNA damage induced ABRO1 upregulation and nuclear translocation, the interaction between ABRO1 and p53 was enhanced after IR treatment (Fig. 6a). Glutathione *S*-transferase (GST) pull-down assays further confirmed the direct binding of ABRO1 to p53 (Supplementary Fig. 7a). Mapping of the binding domain that mediates this interaction revealed that the p53 DBD domain is essential for the binding to the first 200 amino acid residues of ABRO1 (Fig. 6b,c).

USP7 plays a key role in the maintenance of p53 stability. A previous study identified USP7 as a candidate binding partner for ABRO1 (ref. 51). To confirm this, we examined the endogenous interaction between ABRO1 and USP7 in HCT116 p53^+/+^ cells and found that this interaction could be enhanced by DNA damage (Fig. 6d). GST pull-down assays further confirmed the direct binding of ABRO1 to USP7 (Supplementary Fig. 7b). ABRO1 was also found to be associated with USP7 in p53-deficient HCT116 cells (Supplementary Fig. 7c), indicating that ABRO1 may have the ability to bind USP7 in the absence of p53. In immunoprecipitation experiments using cells transfected with various USP7 domains, ABRO1 selectively bound to the C-terminal domain (amino acid residues 561–1,102) of USP7 (Fig. 6e). Deletion analyses also showed that the amino acid residues 215–415 of ABRO1 bind USP7 (Fig. 6f). The critical residues for the interaction between USP7 and p53 are amino acids 53–208 of the USP7 N-terminal domain and amino acids 357–382 of the p53 regulatory region C-terminal domain^22^. Our results indicate that the association between ABRO1, USP7 and p53 occurs at non-overlapping regions of the proteins, suggesting that these three components might form a protein complex. To test this, HCT116 p53^+/+^ cell nuclear extracts were immunoprecipitated with ABRO1-, p53- and USP7-specific antibodies, and the immunoprecipitated proteins were analysed by western blotting. As shown in Supplementary Fig. 7e, ABRO1, p53 and USP7 were co-immunoprecipitated at the endogenous expression levels of the three proteins. In agreement with previous results, immunoprecipitation of p53 and USP7 by ABRO1 was greater after DOX treatment (Supplementary Fig. 7d). Further examination using a sequential immunoprecipitation assay confirmed that ABRO1, p53 and USP7 were present in the same complex (Fig. 6g). We also examined the nuclear co-localization of ABRO1, p53 and USP7 by immunocytochemistry and observed partial nuclear co-localization under normal cellular conditions and full nuclear co-localization after IR treatment (Fig. 6h). These results provide further evidence for the association of these proteins.

### ABRO1 facilitates p53 deubiquitination dependent on USP7

**Figure 7:**
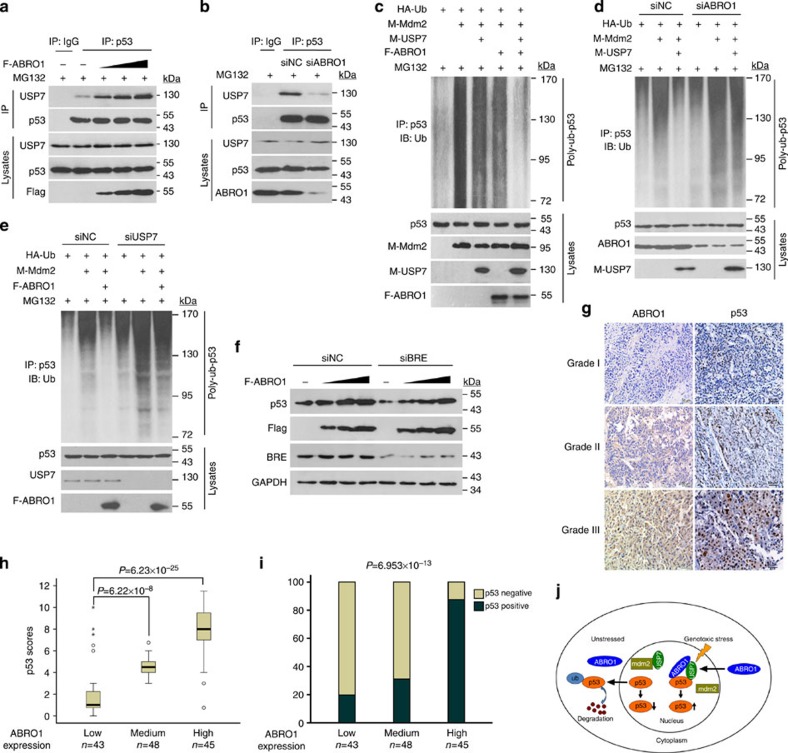
ABRO1 facilitates p53 deubiquitination in a herpes-associated ubiquitin-specific protease (HAUSP)-dependent manner, and its level in primary RCC correlates with expression of p53. HCT116 p53^+/+^ cells transfected with the indicated constructs (**a**) or with siNC or siABRO1 (**b**) were treated with MG132 for 4 h before harvest. Lysates were immunoprecipitated with control IgG or anti-p53 (FL-393) antibody and analysed by immunoblotting with the indicated antibodies. (**c**) HCT116 p53^+/+^ cells co-transfected with the indicated constructs were treated with MG132 for 4 h before harvest. Cell lysates were immunoprecipitated with anti-p53 (FL-393) antibody and analysed by immunoblotting with anti-Ub antibody. HCT116 p53^+/+^ cells transfected with the indicated constructs and siABRO1 (**d**) or siUSP7 (**e**) were treated with MG132 for 4 h before harvest. Cell lysates were immunoprecipitated with anti-p53 (FL-393) antibody and analysed by immunoblotting with the indicated antibodies. (**f**) HCT116 p53^+/+^ cells were co-transfected with Flag-ABRO1 and siBRE or siNC as indicated. Cell lysates were analysed by immunoblotting with the indicated antibodies. (**g**) Representative images showing immunohistochemical staining of ABRO1 and p53 expression in two serial sections of the same RCC tumour from three cases of different histological grades (1–3). Scale bars, 50 μm. (**h**) Box plot of p53 expression in RCC from 136 subjects. The subjects were divided into three groups based on ABRO1 expression scores in the tumours. The samples shown represent low (score 1–4), medium (score 5–8) and high (score 9–12) expression of ABRO1. Outliers are marked with circles and extreme cases are marked with asterisks. The data were analysed using the one-way analysis of variance test with Games–Howell’s correction. Horizontal lines represent the median; the bottom and top of the boxes represent the 25th and 75th percentiles, respectively; and the vertical bars represent the range of data. (**i**) The percentage of p53-positive tumours in the three groups of subjects described in **h**. The data were analysed using Pearson’s *χ*^2^-test. (**j**) Working model of p53 regulation by ABRO1.

Based on the results showing association of ABRO1, p53 and USP7, we presumed that ABRO1 might promote interaction between USP7 and p53, and regulate USP7-dependent deubiquitination of p53. We therefore examined the effect of ABRO1 on the interaction between USP7 and p53. As shown in Fig. 7a, ectopic expression of ABRO1 in HCT 116 p53^+/+^ cells enhanced the interaction between USP7 and p53, whereas knockdown of ABRO1 weakened the interaction between USP7 and p53 in the presence of MG132 (Fig. 7b). In contrast, USP7-p53 interaction was not affected when control siRNA was transfected. To determine whether USP7 affects the interaction between ABRO1 and p53, expression of USP7 in HCT 116 p53^+/+^ cells was knocked down by RNAi. Using Co-IP analysis, we found that knockdown of USP7 expression did not affect the interaction between ABRO1 and p53 (Supplementary Fig. 8a). Consistent with these results, enforced expression of ABRO1 dramatically facilitated USP7-mediated deubiquitination of p53 (Fig. 7c), whereas knockdown of ABRO1 decreased USP7-mediated p53 deubiquitination (Fig. 7d). We further determined whether ABRO1 facilitates p53 deubiquitination in a USP7-dependent manner; as shown in Fig. 7e, knockdown of USP7 impaired ABRO1-mediated p53 stability and deubiquitination. These results indicate that ABRO1 stabilizes and deubiquitinates p53 by increasing the recruitment of USP7 to p53.

As USP7 acts as a specific deubiquitinase for both p53 and MDM2, we determined whether ABRO1 could also facilitate MDM2 deubiquitination via USP7. As shown in Supplementary Fig. 8b, knockdown of ABRO1 in HCT116 p53^−/−^ cells did not affect the ability of USP7 to deubiquitinate MDM2. This result indicates that ABRO1 specifically facilitates USP7-mediated deubiquitination of p53.

### ABRO1 regulates p53 stability independently of the BRISC

ABRO1 is an important component of the BRISC complex, which is composed of four subunits, NBA1, BRE, BRCC36 and ABRO1. To determine whether the effect of ABRO1 on p53 stability is dependent on the BRISC complex, we downregulated NBA1, BRE and BRCC36 protein levels in HCT116 p53^+/+^ cells using siRNAs and examined the effect of this downregulation on p53 levels. As shown in Supplementary Fig. 8c, knockdown of BRE or NBA1 reduced p53 protein level, consistent with a previous report^53^,^54^. However, knockdown of BRCC36 did not affect p53 protein level. We also found that knockdown of BRE did not affect the ability of ABRO1 to regulate p53 stability (Fig. 7f). Moreover, when HCT116 p53^+/+^ cell lysates were immunoprecipitated with p53-specific antibody, USP7 and ABRO1 were co-precipitated, but NBA1, BRE and BRCC36 did not (Supplementary Fig. 8d). Interestingly, under normal conditions, ABRO1 were co-precipitated with NBA1, BRE and BRCC36, but this co-precipitation was not observed after DNA damage (Supplementary Fig. 8e). These results indicate that ABRO1 regulates p53 stability independently of the BRISC complex, especially under DNA damage conditions.

### ABRO1 level in primary RCC correlates with expression of p53

As a very low percentage of renal cell carcinoma (RCC) cases have been found to exhibit p53 mutations^55^ (IARC p53 Database: http://p53.iarc.fr/), we chose RCC as the basis for study of whether ABRO1 expression correlates with p53 expression. We determined the expression levels of ABRO1 and p53 using an RCC tissue microarray containing 136 samples. The resulting staining was classified into three grades, and each sample was graded by pathologists blinded to the identity of the samples (Fig. 7g). We conducted Pearson’s correlation analysis on the data regarding ABRO1 and p53 expression. The results suggest that ABRO1 has a significant positive correlation with p53 expression in carcinomas (Fig. 7h,i). This observation suggests that ABRO1 regulates p53 protein stability.

## Discussion

The protease USP7 acts as a specific deubiquitinase for both p53 and MDM2, and is thus important for p53 regulation. Studies have revealed that p53 and MDM2 compete for the same direct binding site in the N-terminal TRAF (tumour necrosis factor receptor-associated factor) -like domain of USP7. Overexpression of USP7 has been found to result in increased p53 stabilization and therefore increased cell cycle arrest and apoptosis^19^. However, deletion of USP7 does not result in decreased p53 levels. In contrast, p53 levels increase^20^. This is because, in addition to deubiquitination of p53, USP7 also binds to MDM2. Under ordinary conditions, the affinity of USP7 for MDM2 is stronger than that of p53 for MDM2 (refs 21, 22). Under conditions of genotoxic stress, the binding affinity of USP7 and MDM2 is altered through an ataxia telangiectasia mutated (ATM)-dependent phosphorylation, tilting the balance towards p53 stabilization^23^. Thus, USP7 affects p53 stability in two ways: through the deubiquitination of MDM2 and through the deubiquitination of p53. MDMX has also been reported to bind to and be stabilized by USP7, further indicating the complexity of the role of USP7 in regulating p53 stability^23^. Although several proteins are known to regulate the modification, enzymatic activity and selectivity of USP7, the mechanisms underlying this regulation remain completely unexplored. In this paper, we report that ABRO1 acts as a novel positive regulator of p53 stability by tethering USP7 to p53 and promoting p53 deubiquitination and stabilization. Moreover, we also report that ABRO1 is required for efficient induction of p53 by DNA damage and that it inhibits tumour cell proliferation in a p53-dependent manner. Under non-stress conditions, ABRO1 is expressed at a low but consistent level and is mainly distributed in the cytoplasm. This would be beneficial for USP7 targeting of MDM2, enabling the cells to maintain a low level of p53 expression. Under conditions of genotoxic stress, however, the expression of ABRO1 is rapidly induced by DNA damage, and ABRO1 translocates into the nucleus, thereby promoting USP7 targeting of p53 and antagonizing the ubiquitination modification of MDM2 and p53. In this way, ABRO1 could rapidly stabilize and activate p53 (Fig. 7j). These results provide new insight into our understanding of the regulation of p53 stability in response to acute DNA damage.

Several MDM2-binding cofactors have been reported to promote p53 stabilization by directly binding to and inhibiting MDM2. As MDM2 has also been identified as a USP7 target protein, it is intriguing to consider whether the binding of p53 to MDM2 might be affected by ABRO1 and whether ABRO1 might thus influence the ubiquitination and degradation of p53. Here we show that ABRO1 does not affect the stability of MDM2 protein or the binding of p53 to MDM2. Moreover, p53 was found to be upregulated in MDM2^−/−^p53^−/−^ double-deficient MEFs after co-transfection of both p53 and ABRO1, indicating that ABRO1-dependent p53 stabilization is made possible by enhanced p53-USP7 interaction without effects on p53-MDM2 interaction and MDM2 stability. Our results therefore suggest that the ABRO1 has a critical role in determining the specific regulation of ligases by USP7. It has been shown that ABRO1 is a scaffold protein that recruits polypeptides to facilitate assembly of the BRISC DUB enzyme. The DUB activity of the BRISC complex is exclusively directed against Lys63-linked polyubiquitin. K63-linked polyubiquitin has a non-proteolytic role and regulates protein function, subcellular localization and protein–protein interactions. Previous studies reported that increases in ABRO1 levels lead to significant reduction in Lys63-linked ubiquitination of specific protein targets^48^. In the present work, we show that knockdown of BRCC36, the catalytic subunit of the BRISC DUB enzyme, does not affect p53 protein levels. Moreover, knockdown of BRE did not affect the ability of ABRO1 to regulate p53 stability, and NBA1, BRE and BRCC36 were not co-immunoprecipitated by p53-specific antibody, indicating that ABRO1 regulates p53 stability independently of the BRISC complex.

ABRO1 also acts as a tumour suppressor by regulating p53 stability and function. ABRO1 overexpression stabilized p53 and strongly inhibited the growth of wild-type p53-expressing carcinoma cells, but had no effect on p53-null H1299 and HCT 116 cells. These results suggest that inhibition of cell growth by ABRO1 upregulation is p53 dependent. Moreover, we provided evidence that overexpression of ABRO1 also causes p53-dependent G1 cell cycle arrest, and that decreased ABRO1 expression promotes cellular transformation. These results suggest that ABRO1 may suppress tumourigenesis. The potential tumour-suppressive role of ABRO1 may be clinically relevant, because ABRO1 protein levels are significantly reduced in several cancer tissues, including liver, kidney, breast and thyroid gland cancers, and higher ABRO1 expression level correlates with better survival in HCC patients. Furthermore, we found that decreased ABRO1 levels in primary RCC were correlated with the downregulation of p53 protein expression. As a very low percentage of RCC cases have been found to have p53 mutations, decreased expression of ABRO1 could be another mechanism leading to inhibition of p53 function in RCC. These findings suggest that ABRO1 may function as a tumour suppressor.

Taken together, the results presented in this paper demonstrate that ABRO1 plays an important role in the USP7-p53 regulatory loop. ABRO1 is upregulated following DNA damage and acts to selectively stabilize p53 by facilitating the interaction of p53 with the deubiquitinase USP7. Our findings also support the idea that ABRO1 plays a role in human tumour growth suppression by regulating p53 and suggest that it might be possible to develop therapeutic agents that target the interaction between ABRO1 and p53.

## Methods

### Histopathologic analyses with immunohistochemistry

The majority of tissues used in this work were purchased from Outdo Biotech (Shanghai, China; http://www.superchip.com.cn/). These include hepatic carcinoma (HLiv-HCC060PG-01, HLiv-HCC150CS-01 and OD-CT-DgLiv01-012), breast carcinoma (HBre-Dsuc060CS-01 and OD-CT-RpBre03-005), RCC (HKid-CRCC060PG-01, HKid-CRCC150CS-01 and OD-CT-UrKid03-003) and thyroid gland carcinoma (OD-CT-EdThy03-002). For survival analysis, two independent cohorts of HCC patients were used. These were Cohort 1, consisting of 90 HCC patients whose tissues were obtained from Outdo Biotech (HLiv-HCC180Sur-01), and Cohort 2, comprising 161 patients from Affiliated XiNan Hospital of the Third Military Medical University (Chongqing, China). Cohort 2 liver tissue samples were obtained from patients during surgery; details regarding these tissue samples are shown in Supplementary Data 1. Surgically removed tissues were quickly frozen in liquid nitrogen until analysis. Cohort 2 samples were collected with informed consent from patients, and the experiments were approved by the Ethics Committee of the Third Military Medical University. Immunohistochemical staining with ABRO1 (dilution 1:200) or p53 (dilution 1:500) antibodies was carried out using the IHC Select HRP/DAB kit (cat. DAB50, Millipore). Histological staining was assessed by pathologists blinded to the origin of the samples. Staining intensity and area were assessed using the widely accepted German semi-quantitative scoring system^56^. The final immunoreactivity score was determined by multiplying the intensity score by the extent of the score of stained cells and ranged from a minimum score of 0 to a maximum score of 12. We defined a score of 0 as negative and scores of 1–12 as positive.

### Plasmids and antibodies

The human full-length ABRO1 was amplified by PCR from the human liver complementary DNA and cloned into the pFLAG-CMV2 vector (Sigma, St Louis, MO) or pcDNA3.1 vector (Invitrogen), or pBPLV lentiviral vector (Invitrogen). The resulting plasmids were sequenced and used for all subsequent cloning. Expression plasmids HA-p53, GST-p53, HA-ub, Myc-MDM2 and PIG3-Luc, p21-Luc, Bax-Luc reporters were kindly provided by Dr Xueming Zhang (China National Center of Biomedical Analysis, Beijing, China). The series of plasmids encoding deletion mutants of p53 were a gift from Dr Linqiang Zhang (Beijing Institute of Radiation Medicine, Bejing, China). Myc-USP7 expression plasmid was kindly provided by Dr A.G. Jochemsen (Department of Molecular Cell Biology, Leiden University Medical Center, Leiden, The Netherlands). GST-USP7 plasmid was generated by PCR amplification and cloned into pGEX-4T-2 vector (Amersham). GFP-MDM2 was generated by PCR amplification and cloned into pEGFP-N1 vector (Clontech). The series of plasmids encoding deletion mutants of ABRO1 or USP7 was constructed by inserting fragments generated by PCR and cloned into pFLAG-CMV2 vector or pcDNA3.1 vector. siRNA-resistant ABRO1 construct (FLAG-CMV2) was generated by introducing four base-pair mutations into the ABRO1 cDNA without altering the encoded protein sequence using a Quickchange mutagenesis kit (Stratagene). The Lentivirus vector pLKO.1-ABRO1-shRNA and pLKO.1-ABRO1-scramble were provided by Dr Junjie Chen (Department of Experimental Radiation Oncology, University of Texas M. D. Anderson Cancer Center, Houston, Texas, USA).

The p53 monoclonal antibody (DO-1) (1:1,000), p53 polyclonal antibody (FL-393) (1:1,000) and HA(F-7) (1:500), Myc(9E10) (1:1,000), p21 (1:500), USP7(C-2) (1:1,000), c-myc-HRP(9E10) (1:1,000), MDM2(SMP4) (1:500), BRE(C-6) (1:2,000) and MERIT40(S-12) (1:1,000) antibodies were purchased from Santa Cruz Biotechnology. The BRCC36(EPR4366) (1:1,000) antibody and the FAM175B (ABRO1) (1:500) mouse polyclonal antibody were purchased from Abcam Inc. The antibody against Flag(M2) (1:5,000) was purchased from Sigma. The antibody against GAPDH(10494-1-AP) (1:2,000) was purchased from Biotech. The ABRO1 rabbit monoclonal antibody (1:1,000) was prepared in our lab.

### Cell culture transfection and luciferase assays

293T, SKNSH, H1299, MCF-7, HepG2, HeLa, HT-29, BT474, A549, MEF p53^−/−^MDM2^−/−^, L02, HCT116 p53^+/+^ and HCT116 p53^−/−^ cells were cultured in DMEM or RPMI1640 supplemented with 10% fetal bovine serum (Gibco) and maintained at 37 °C in a humidified atmosphere of 5% CO_2_. Transfection was performed using Lipofectamin2000 according to the manufacturer’s protocol. Stably transfected clones were generated by selection against neomycin with G418. Luciferase assays were performed as described previously^57^,^58^. Luciferase activities were expressed as fold induction relative to the values obtained from control cells. The results represent the mean of at least three independent transfection experiments, each carried out in duplicate.

### Immunoprecipitation and immunoblotting

Cells were collected and lysed for 30 min on ice in lysis buffer (50 mM Tris–HCl (pH 7.4), 150 mM NaCl, 1 mM EDTA, 1% TritonX-100) supplemented with protease inhibitor cocktail (Roche, Basel, Switzerland). Soluble cell lysates were incubated with 2 μg of indicated antibodies for 2 h at 4 °C, followed by incubation at 4 °C with protein A/G agarose beads overnight. Unbound proteins were removed by washing four times with lysis buffer. The elution buffer (50 mM Tris–HCl (pH 7.4), 900 mM NaCl, 1 mM EDTA, 1% Triton X-100) was used to remove the immunoprecipitates from agarose beads for sequential Co-IP. Following SDS–PAGE, immunoprecipitated proteins were transferred onto polyvinylidene difluoride membranes (Amersham Life Science, Buckinghamshire, England) and probed with various antibodies. The enhanced chemiluminescence system (Santa Cruz Biotechnology, Santa Cruz, CA) was used for detection. Western blottings were quantified using ImageJ software (rsbweb.nih.gov/ij/). All original western blottings are shown in Supplementary Fig. 9.

### RNA interference

The target sequences for the siRNA against ABRO1 and nonspecific siRNA were as follows: siABRO1-1, 5′-AUU CAC UAU UAG AAG GCU CUG-3′^59^; siABRO1-2, 5′-AGG UAU AAU CAG AGG AUA UTT-3′; siABRO1-3, 5′-GGC AGA AGU GAA CAA AUU ATT-3′; and siNC, 5′-CUGGACUUCCAGAAGAACAUC-3′. The siRNA duplex sequences used for the depletion of USP7, BRCC36, MERIT40/NBA1 and BRE were as previously described^60^.

ABRO1 shRNA sequence was shuttled into lentiviral expression vector pLKO.1. The pLKO.1-shRNA-ABRO1 was co-transfected with pLP1, pLP2 and VSVG into 293T cells. After 48 h, the recombinant lentiviruses were packaged and purified^61^,^62^,^63^.

### FACS analysis

For cell cycle analysis, cells were pulsed for 1 h with BrdU before being harvested, fixed, stained with anti-BrdU fluorescein isothiocyanate and propidium iodide, sorted by flow cytometry and analysed for DNA content, as previously described^64^.

For apoptosis analysis, cells were collected and cell apoptosis was determined by annexin V (Sigma) staining using flow cytometry, as previously described^65^.

### Pull-down assays

GST-p53 or GST-USP7 plasmids were introduced into *Escherichia coli* BL21 strains, and the recombinant proteins were induced by the addition of 0.5 mM isopropyl-β-D-thiogalactoside at 37 °C for 6 h. HEK293 cells expressing Flag-ABRO1 were harvested with RIPA buffer (50 mM Tris–HCl (pH 7.4), 150 mM NaCl, 1% NP-40, 0.5% dithiothreitol, 0.1% SDS). Cell lysates were treated with DNase I (TaKaRa, Japan) for 30 min at 37 °C to remove the genomic DNA contamination before immunoprecipitation with Anti-Flag M2 agarose (Sigma). After thoroughly washing, specifically bound proteins were incubated with GST, and GST–p53 or GST-USP7 fusion protein bound to Sepharose beads in 1 ml of binding buffer (20 mM Tris–HCl, (pH 8.0), 150 mM NaCl, 1 mM EDTA, 10% glycerol and 0.1% Nonidet P-40) at 4 °C for 4 h. The beads then were washed and eluted in 20 μl of 2 × SDS–PAGE sample buffer and detected by immunoblotting.

### Cytoplasmic and nuclear protein fractionation

HCT116 cells were transfected with the indicated constructs. After 48 h, the cells were treated with MG132 (20 μM) for 6 h. Subsequently, the cells were collected by centrifugation for 5 min at 900 r.p.m. and washed three times with cold PBS. Cytoplasmic and nuclear fractions were separated using NE-PER Nuclear and Cytoplasmic Extraction Reagents (Sigma), following the manufacturer’s procedures.

### RNA isolation and PCR analysis

Total RNA was isolated using TRIZOL reagent (Invitrogen) in accordance with the manufacturer’s instructions, and the first-strand cDNA was synthesised with the Thermo Script RT–PCR kit (Invitrogen) in accordance with the manufacturer’s instructions. Applied Biosystems 7500 Real-Time PCR system software was used for real-time PCR analysis. The analysis was performed with SYBR-green I fluorescence (Applied Biosystems). Quantification of glyceraldehyde 3-phosphate dehydrogenase (GAPDH) mRNA (as an internal control for gene expression in the cells) was performed with TaqMan Human GAPDH Control Reagents (with VIC Probe, Applied Biosystems). For semi-qRT–PCR analysis, RT–PCR was performed with a Platinum qRT–PCR kit (Invitrogen) in accordance with the manufacturer’s instructions. PCR products were separated on 1.5% agarose gel. The primers used in this study were as follows: ABRO1-forward-primer: 5′-TCCATAACCATCAGCCTTGTTC-3′; ABRO1-reverse-primer: 5′-CCCAATGACTTTCTTTCTCCGA-3′; GAPDH-forward-primer: 5′-GGAGCGAGATCCCTCCAAAAT-3′; GAPDH-reverse-primer: 5′-GGCTGTTGTCATACTTCTCATGG-3′; p53-forward -primer: 5′-CAGCACATGACGG AGGTTGT-3′; p53-reverse-primer: 5′-TCATCCAAATA CTCCACACGC-3′; p21-forward-primer: 5′-TGTCCGTCAGAACCCATGC-3′; p21-reverse-primer: 5′-AAAGTCGAAGTTCCATCGCTC-3′; Mdm2-forward-primer: 5′-GAATCATCGGACTC AGGTACATC-3′; and Mdm2-reverse-primer: 5′-TCTGTCTCACTAATTGCTCTCCT-3′.

### Clone and methylcellulose clone-formation assays

Clone formation was assessed by plating 1 × 10^3^ cells per 60-mm dish. After 14 days of cultivation, crystal violet staining was used to visualize clones, and clones that were 2 mm or greater in size were scored. The methylcellulose clone-formation assay was performed as previously described^63^. The cells were plated in 1% methylcellulose diluted with RPMI 1640 for 2 weeks. The clones were counted under a light microscope.

### Tumourigenicity assays in mice for xenograft tumour study

To examine the tumourigenicity of HCT-116 cells *in vivo*, one million HCT-116 cells with or without constitutive expression of ABRO1 were injected into 4- to 6-week-old nude mice (*n*=10) obtained from the Beijing Institute of Radiation Medicine Animal Center (Beijing, China). Xenograft tumour growth was monitored by calliper measurement at various time points. After 4 weeks, the mice were killed and the tumours were removed. All animal studies were approved by the Animal Care Committee of Beijing Institute of Radiation Medicine. The animals received humane care according to the criteria outlined in the ‘Guide for the Care and Use of Laboratory Animals’ prepared by the National Academy of Sciences and published by the National Institutes of Health.

### Immunofluorescence assays

For the p53 translocation assay, HCT116 p53^+/+^ cells were seeded on glass coverslips and transfected as indicated. Forty-eight hours after transfection, the cells were treated with MG132 (20 μM) for 6 h before fixation. The cells were then fixed in 4% paraformaldehyde for 15 min at room temperature and incubated with anti-p53 (1:200), anti-ABRO1 (1:200) or anti-Flag (1:1,000) antibodies overnight. The cells were then washed with PBS, incubated with secondary antibodies conjugated with Alexa Fluor 488, Alexa Fluor 594, or Alexa Fluor 350, and stained with 4',6-diamidino-2-phenylindole at room temperature. For the ABRO1 translocation assay, HCT116 p53^+/+^ and A549 cells were seeded on glass coverslips and treated as indicated. After 12 h, the cells were fixed in 4% paraformaldehyde for 10 min at room temperature and stained using standard protocols.

### Deubiquitination of p53

HCT116 cells were transfected with the indicated constructs. Twenty-four hours after transfection, the cells were treated with the proteasome inhibitor MG132 (20 μM) before being harvested and lysed in lysis buffer. Ubiquitinated p53 was immunoprecipitated with p53 (FL-393) antibody and immunoblotted with anti-Ub antibody or anti-p53 antibody (DO-1).

### Statistics

Data are presented as the mean±s.d. Statistical comparisons between experimental groups were analysed using Student’s *t*-test. The correlation between ABRO1 expression and p53 expression was determined by Pearson’s correlation test. We defined survival time as the time from the date of surgery to the subject’s death from cancer. Subjects who were alive at the last follow-up were recorded at the last follow-up date. Survival curves were derived from Kaplan–Meier estimates. For analysis of the survival of HCC patients, the log-rank test in SPSS 19.0 was used with the *P*-values indicated. All statistical tests were two-sided, and *P*-values <0.05 were considered to be statistically significant.

## Author contributions

X.Y., J.Z. and M.C. conceived and designed the project, and wrote the manuscript. J.Z., M.C., W.X., Y.Z. and X.W. designed and conducted the experiments, including immunoprecipitation, RT–PCR, western blotting and immunofluorescence assays. J.D. obtained clinical samples and performed the immunohistochemistry analysis. C.L. and M.Y. constructed stable cell lines. J.Z., M.C. and Z.G. carried out animal experiments. J.Z., M.C. and C.G. contributed to the preparation of the cDNA vector constructs. J.Z. and Y.Y. performed the statistical analyses.

## Additional information

**How to cite this article:** Zhang, J. *et al.* ABRO1 suppresses tumourigenesis and regulates the DNA damage response by stabilizing p53. *Nat. Commun.* 5:5059 doi: 10.1038/ncomms6059 (2014).

## Supplementary Material

Supplementary Figures and Tables Supplementary Figures 1-9 and Supplementary Tables 1-2

Supplementary Data 1 Characteristics of Cohort 2 HCC patients used for survival analysis

## Figures and Tables

**Table 1:** ABRO1 levels in tumour samples.

**Tumour samples**	**Samples with decreased ABRO1 expression**	
	**Ratio of decreased samples**	Average tumour/adjacent in decreased samples*
Hepatic cellular cancer	256/310 (83%)	0.39±0.21
Thyroid cellular cancer	21/31 (68%)	0.22±0.13
Breast cellular cancer	43/61 (70%)	0.61±0.17
Renal cellular cancer	97/136 (71%)	0.53±0.19

^*^Data of tumour/adjacent are shown as mean±s.d. (*n*=samples with deregulated ABRO1 expression).
