# Supplementary material for: ABRO1 suppresses tumourigenesis and regulates the DNA damage response by stabilizing p53
Source: Nat Commun. 2014 Oct 6;5:5059. doi: 10.1038/ncomms6059 (PMC4205886; doi:10.1038/ncomms6059)
Supplement: Supplementary Figures and Tables — Supplementary Figures 1-9 and Supplementary Tables 1-2 [file ncomms6059-s1.pdf]

Supplementary Information

ABRO1 suppresses tumourigenesis and regulates the DNA damage response by stabilizing p53

Jianhong Zhang, *et al.*

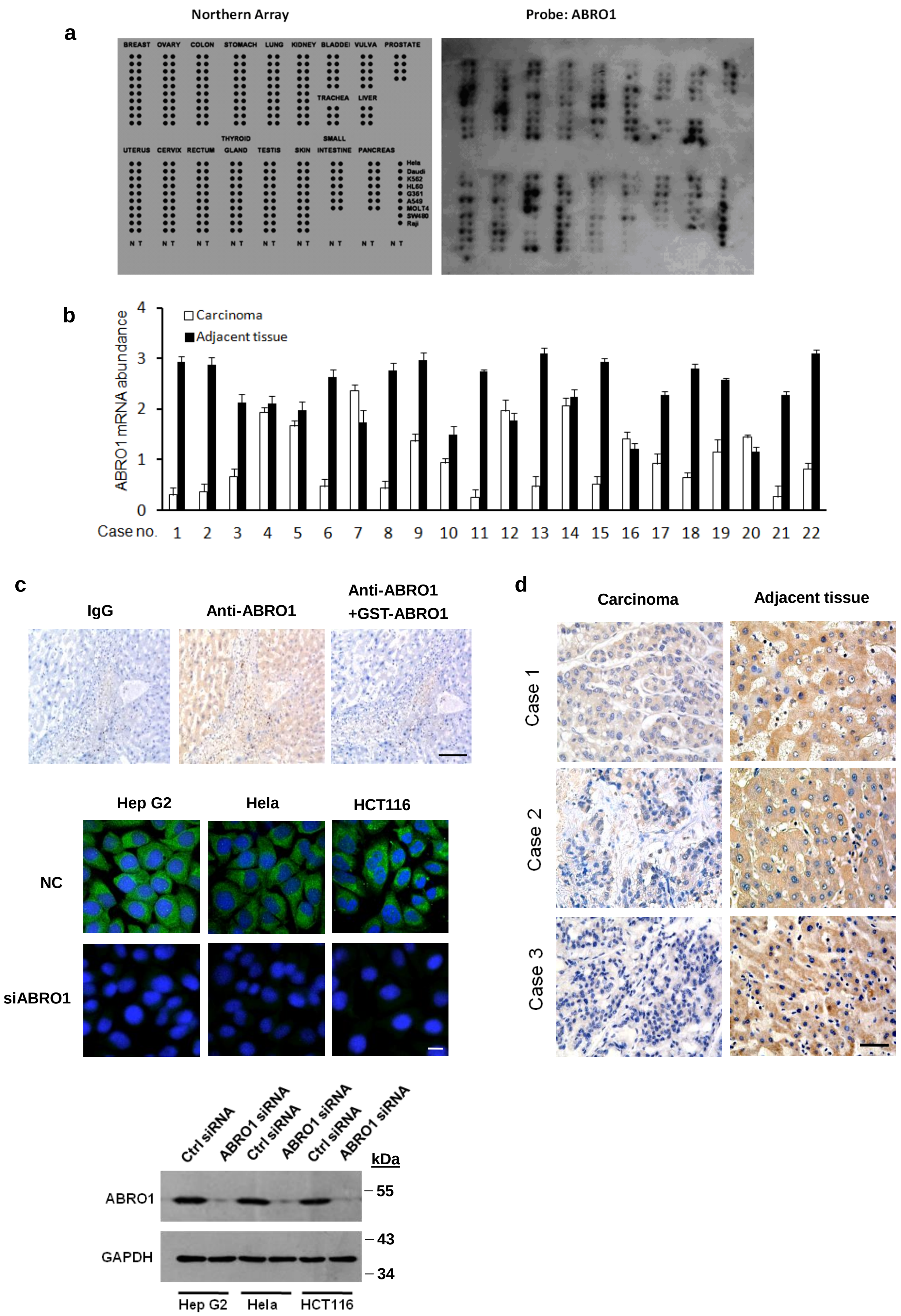
a
Northern Array
Probe: ABRO1
BREAST
OVARY
COLON
STOMACH
LUNG
KIDNEY
VULVA
PROSTATE
TRACHEA
LIVER
THYROID GLAND
SMALL INTESTINE
UTERUS
CERVIX
RECTUM
TESTIS
SKIN
PANCREAS
Hela
Daudi
K562
HL60
G361
A549
MOLT4
SW480
Raji
N T
b
ABRO1 mRNA abundance
Carcinoma
Adjacent tissue
0
1
2
3
4
Case no.
1 2 3 4 5 6 7 8 9 10 11 12 13 14 15 16 17 18 19 20 21 22
c
IgG
Anti-ABRO1
Anti-ABRO1 +GST-ABRO1
Hep G2
Hela
HCT116
NC
siABRO1
Ctrl siRNA
ABRO1 siRNA
Ctrl siRNA
ABRO1 siRNA
Ctrl siRNA
ABRO1 siRNA
kDa
ABRO1
GAPDH
55
43
34
Hep G2
Hela
HCT116
d
Carcinoma
Adjacent tissue
Case 1
Case 2
Case 3

Supplementary Figure 1. The differential expression pattern of ABRO1 in human multiple tumour tissues and corresponding normal tissues. (a) The radioactived *ABRO1*-specific probe was hybridized onto an array containing 154 cDNA pairs of samples derived from multiple human tumours and corresponding normal tissue from individual patients (Cancer Profiling Array Ⅱ, Clontech). N = normal tissue sample. T = tumour tissue sample. **(b)** ABRO1 mRNA extracted from 22 HCC patients was detected with ABRO1 and GAPDH primers. Data are shown as means ± s.d. of three independent experiments. **(c)** The validation of the specificity of the antibody to ABRO1. Upper panels, Immunohistochemical staining for hepatic cancer specimens incubated with IgG or *ABRO1*-specific antibody. To validate the specificity, monoclonal antibody to ABRO1 was pre-incubated with recombinant GST-ABRO1 protein for 1 h prior to applying to tissue. Scale bar represents 50μm. Middle panels, immunofluorescence staining for ABRO1 in HepG2, HeLa and HCT116 cells transfected with control or ABRO1 siRNA. Scale bar represents 10μm. Bottom panels, Western blot analysis of the lysates from the cells as in middle. **(d)** Immunohistochemical staining for hepatic cancer specimens incubated *ABRO1*-specific antibody. Representative pictures were selected to show the alterations in the expression of ABRO1. Scale bar represents 35μm.

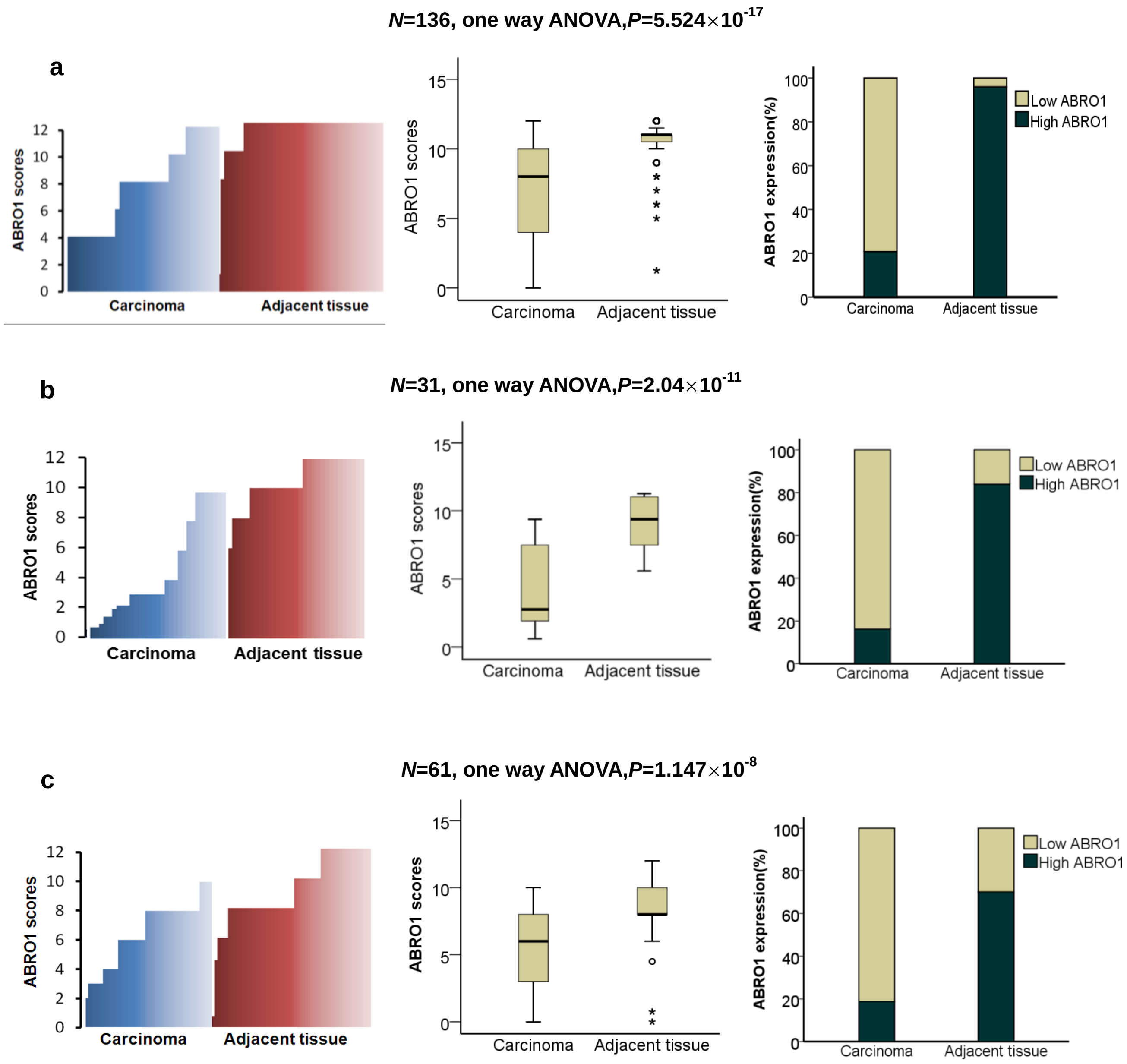

Supplementary Figure 2. The downregulation of ABRO1 in different kinds of tumours. Immunohistochemical analyses were performed to determine the expression of ABRO1 in renal cell cancer tissues **(a)**, thyroid gland cancer tumour tissues **(b)**, breast cancer tissues **(c)** and normal adjacent tissue samples. *P*<0.01, one-way ANOVA.

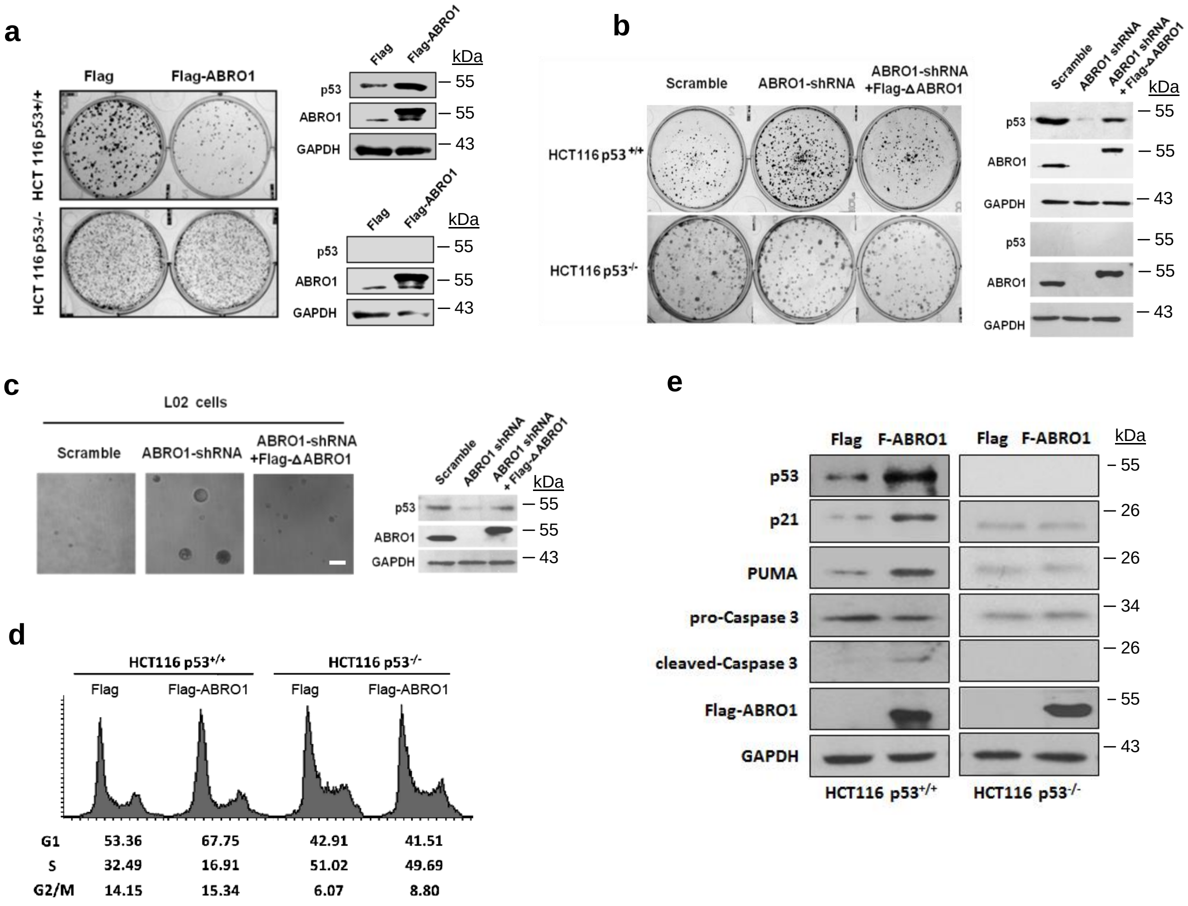

Supplementary Figure 3. ABRO1 suppress tumourigenesis in a p53-dependent manner. **(a)** HCT116 p53$^{+/+}$ and HCT116 p53$^{-/-}$ cells were transfected with pCMV-Flag-ABRO1 or pCMV-Flag vectors, and the number of G418-resistant clones was determined (Left). Lysates of G418-resistant cells were subjected to western blot with anti-p53, anti-ABRO1 and anti-GAPDH antibodies (Right). **(b)** HCT116 p53$^{+/+}$ and HCT116p53$^{-/-}$ cells were infected with control pBPLV lentivirus or pBPLV lentivirus encoding shRNA specific to ABRO1 or pBPLV lentivirus encoding ABRO1 shRNA and RNAi-immune ABRO1. GFP-positive cells were selected by FACS and clones were counted 2 weeks after initial plating (Left). Lysates were subjected to western blot with anti-p53, anti-ABRO1 and anti-GAPDH antibodies (Right). **(c)** L02

cells were infected with control pBPLV lentivirus or pBPLV lentivirus encoding shRNA specific to ABRO1 or pBPLV lentivirus encoding ABRO1 shRNA and RNAi-immune ABRO1, and plated into soft agar. GFP-positive cells were selected by FACS and clones were counted 2 weeks after initial plating (Left). Scale bar represents 200μm. Lysates were subjected to western blot with anti-p53, anti-ABRO1 and anti-GAPDH antibodies (Right). **(d)** HCT116 $p53^{+/+}$ and HCT116 $p53^{-/-}$ cells were transfected with pCMV-Flag-ABRO1 or pCMV-Flag control vectors, and the G418-resistant cells were selected. Cells were pulsed for 1 h with BrdU before being harvested, fixed, stained with anti-BrdU FITC and propidium iodide (PI), sorted by flow cytometry and analyzed for DNA content. **(e)** Proteins extracted from tumours of nude mice were subjected to western blot with anti-p53, anti-p21, anti-PUMA, anti-Caspase 3, anti-Flag and anti-GAPDH antibodies.

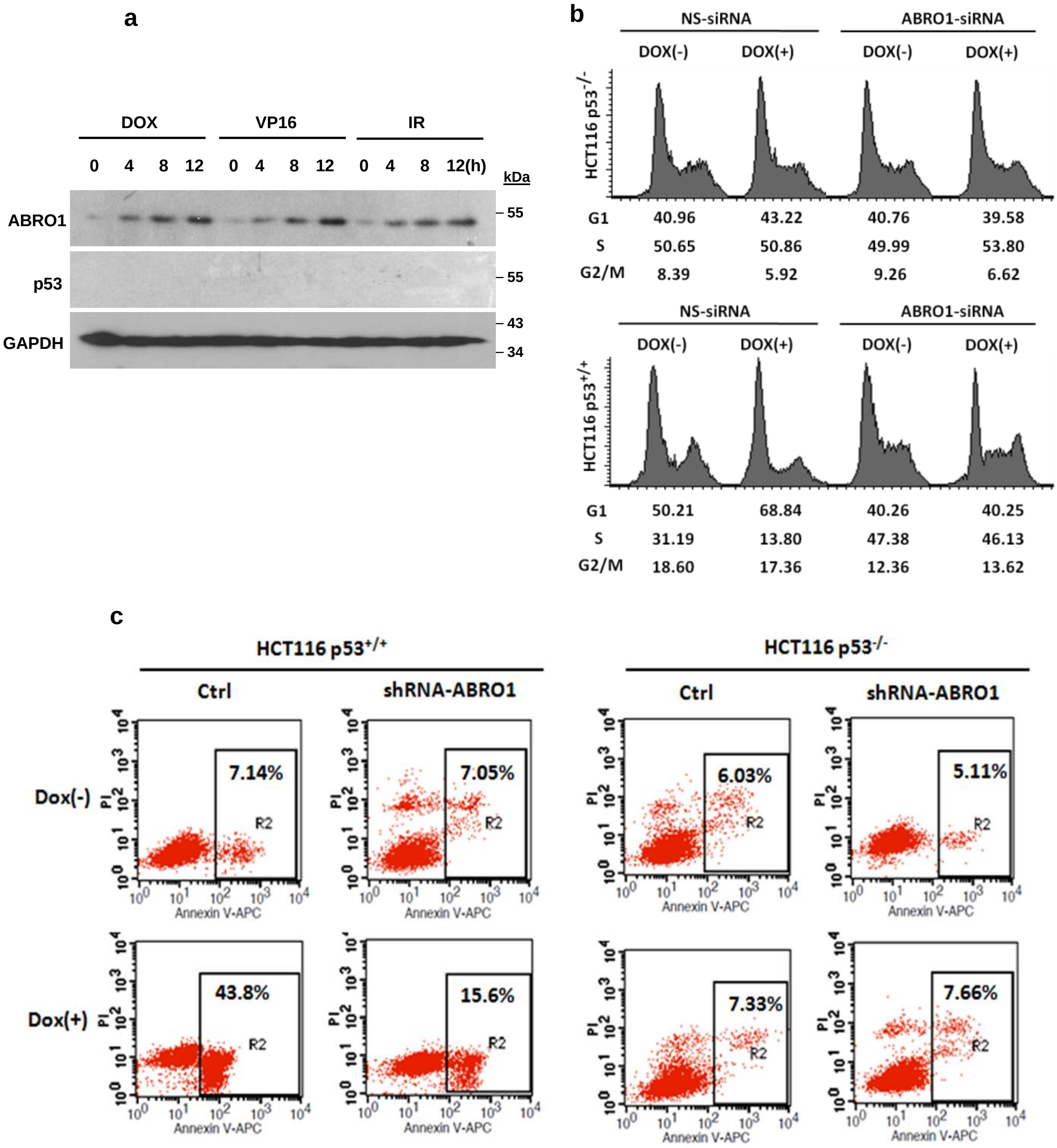

Supplementary Figure 4. ABRO1 increase apoptosis via p53 in response to DNA damage. (a) HCT116 $p53^{-/-}$ cells were treated with DOX, VP16 and IR as indicated times, protein were extracted and subjected to western blot. **(b)** HCT116 cells were transfected with siABRO1-1 or siNC. 48 h after transfection, cells were treated with 2

μg/ml DOX for 8 h before harvested and cell-cycle profiles were determined by FACS as described in Supplementary Figure 3d. **(c)** HCT116 cells were infected with control pBPLV lentivirus or pBPLV lentivirus encoding shRNA specific to ABRO1, and treated with 2 μg/ml DOX for 8 h. Cell apoptosis was examined by FACS.

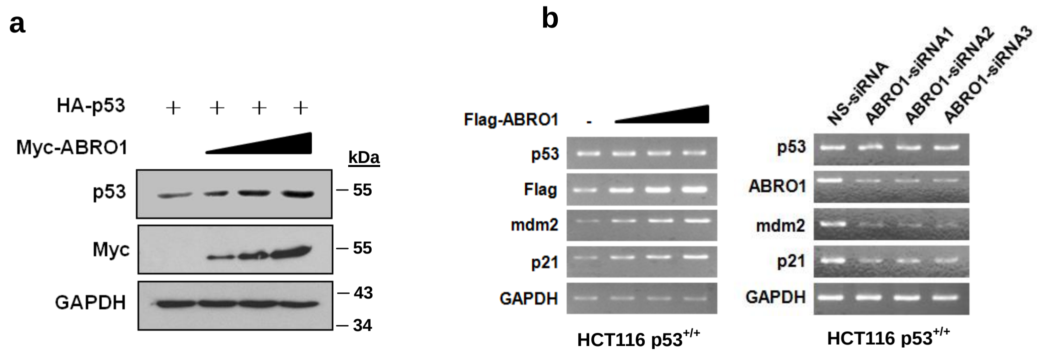

Supplementary Figure 5. ABRO1 effects p21, MDM2 but not p53 mRNA level. (a) HCT116 $p53^{-/-}$ cells were cotransfected with HA-p53 and Myc-ABRO1 expression constructs, after 48 hours, the lysates were analyzed by immunoblotting. **(b)** HCT116 $p53^{+/+}$ cells were transfected with ABRO1 expression vector (left) or ABRO1 siRNA (right) as indicated, after 48 hours, total RNAs were extracted and analyzed by RT-PCR.

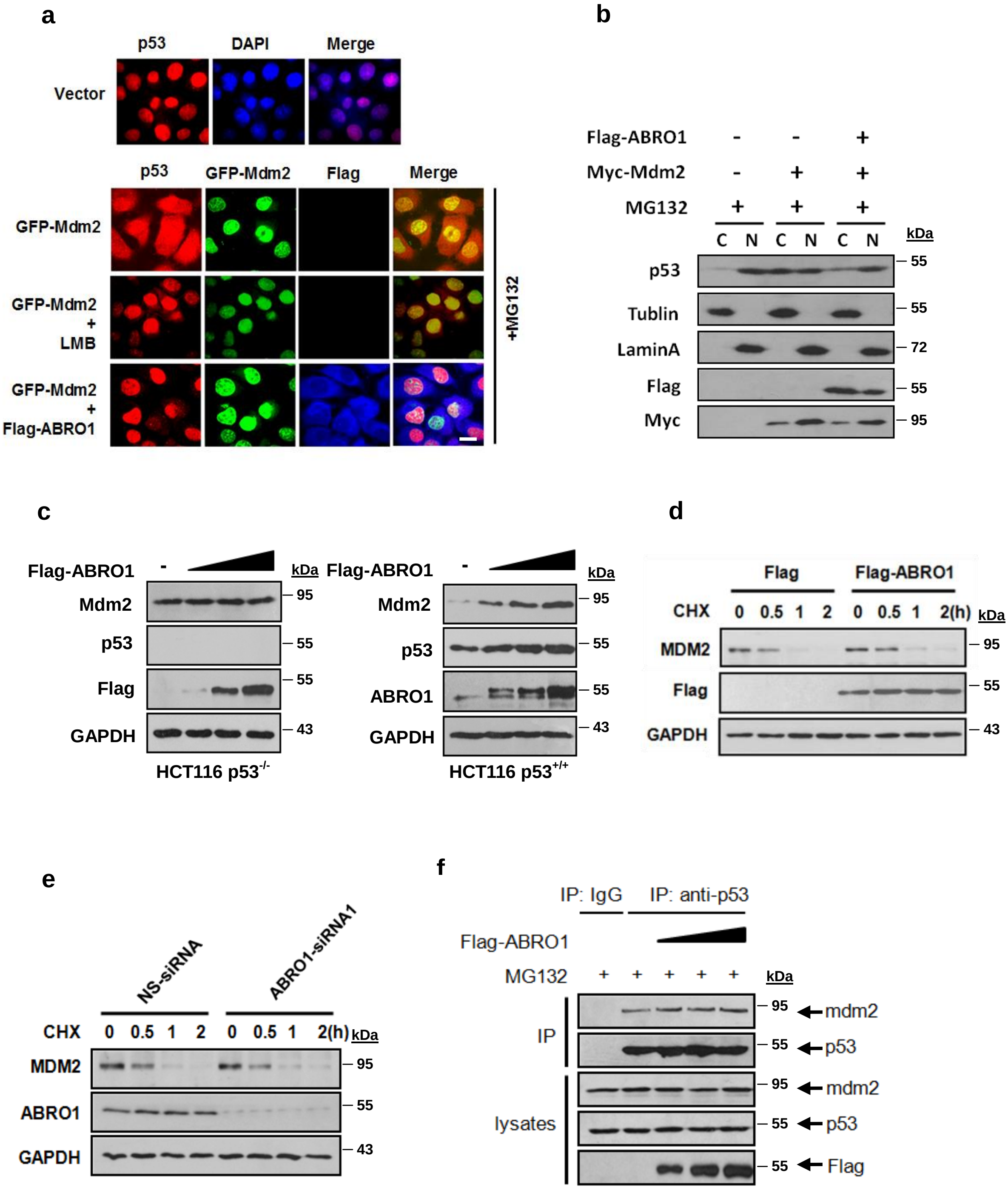

Supplementary Figure 6. ABRO1 counteracts Mdm2 induced p53 cytoplasmtranslocation. **(a)** HCT 116 p53$^{+/+}$ cells were seeded onto small slide glass coverslips placed in 24 culture plates, and transfected with the indicated

constructs. After 36 h, cells were treated with 20 μM MG132 for 6 h before fixed with 4% PFA, immunochemistry assay was performed with p53 and ABRO1 antibodies. Leptomycin B was used as a positive control. Scale bar represents 10 μm. **(b)** HCT 116 p53$^{+/+}$ cells were treated as described in **a**, cell fractionation assay separating cytoplasmic from nuclear proteins was performed and the proteins were immunoblotted with the indicated antibodies. **(c)** HCT116 p53$^{-/-}$ cells or HCT116 p53$^{+/+}$ cells were transfected with Flag-ABRO1 expression construct, after 24 hours, the lysates were analyzed by immunoblotting as indicated. **(d, e)** HCT116 p53$^{-/-}$ cells treated with CHX (1μg/μl) at the indicated time after transfection with Flag-ABRO1 construct or ABRO1 siRNA. Proteins were extracted and subjected to western blot. **(f)** HCT116 p53$^{+/+}$ cells transfected with increasing concentrations of plasmid encoding Flag–ABRO1 were treated with MG132 for 4 hours before harvest. Lysates were immunoprecipitated with control IgG or anti-p53 (FL-393) antibody and analyzed by immunoblotting with indicated antibodies.

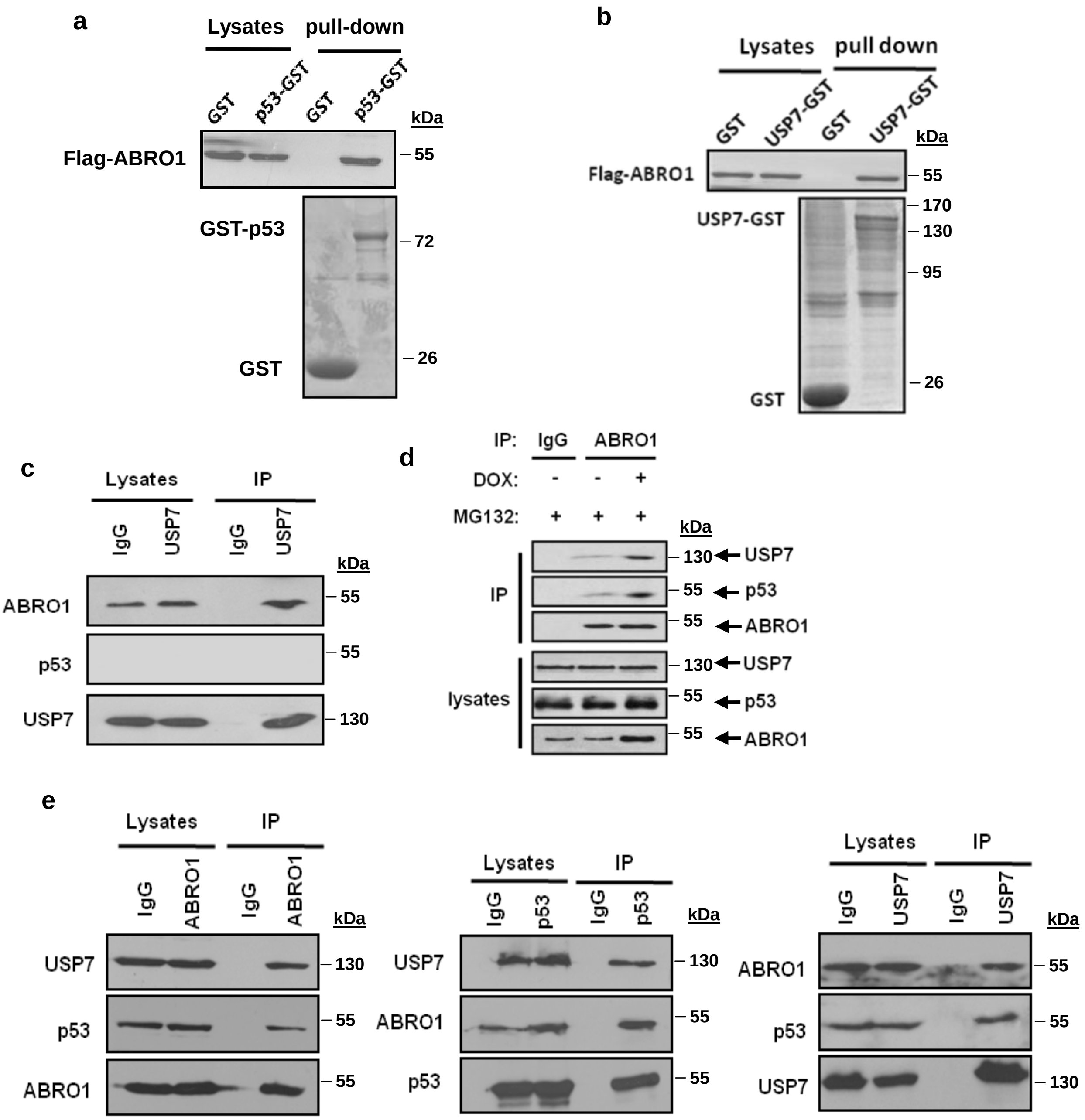

Supplementary Figure 7. ABRO1 directly binds p53 or USP7 and forms a Complex. (a) Purified ABRO1 was incubated with GST or GST-p53 coupled to GST-Sepharose. Proteins retained on sepharose were then blotted with the indicated antibodies. **(b)** Purified ABRO1 was incubated with GST or GST-USP7 coupled to GST-Sepharose. Proteins retained on sepharose were then blotted with the indicated

antibodies. **(c)** Lysates of HCT116 p53$^{-/-}$ cells were subject to immunoprecipitation with control IgG and anti-USP7 antibody. The immunoprecipitates were then blotted with the indicated antibodies. **(d)** The interaction between extent of immunoprecipitation of p53 and USP7 by ABRO1 was greater after DOX treatment than control condition. HCT116 p53$^{+/+}$ cells were treated with DOX for 12h, and then treated with MG132 for 6h before harvested. Lysates were extracted for immunoprecipitation assay with IgG or anti-ABRO1 antibody. The immunoprecipitates were then blotted with the indicated antibodies. **(e)** HCT116 p53$^{+/+}$ cell lysates were subjected to immunoprecipitation with control IgG, anti-p53, anti-USP7 or anti-ABRO1 antibodies. The immunoprecipitates were then blotted with the indicated antibodies.

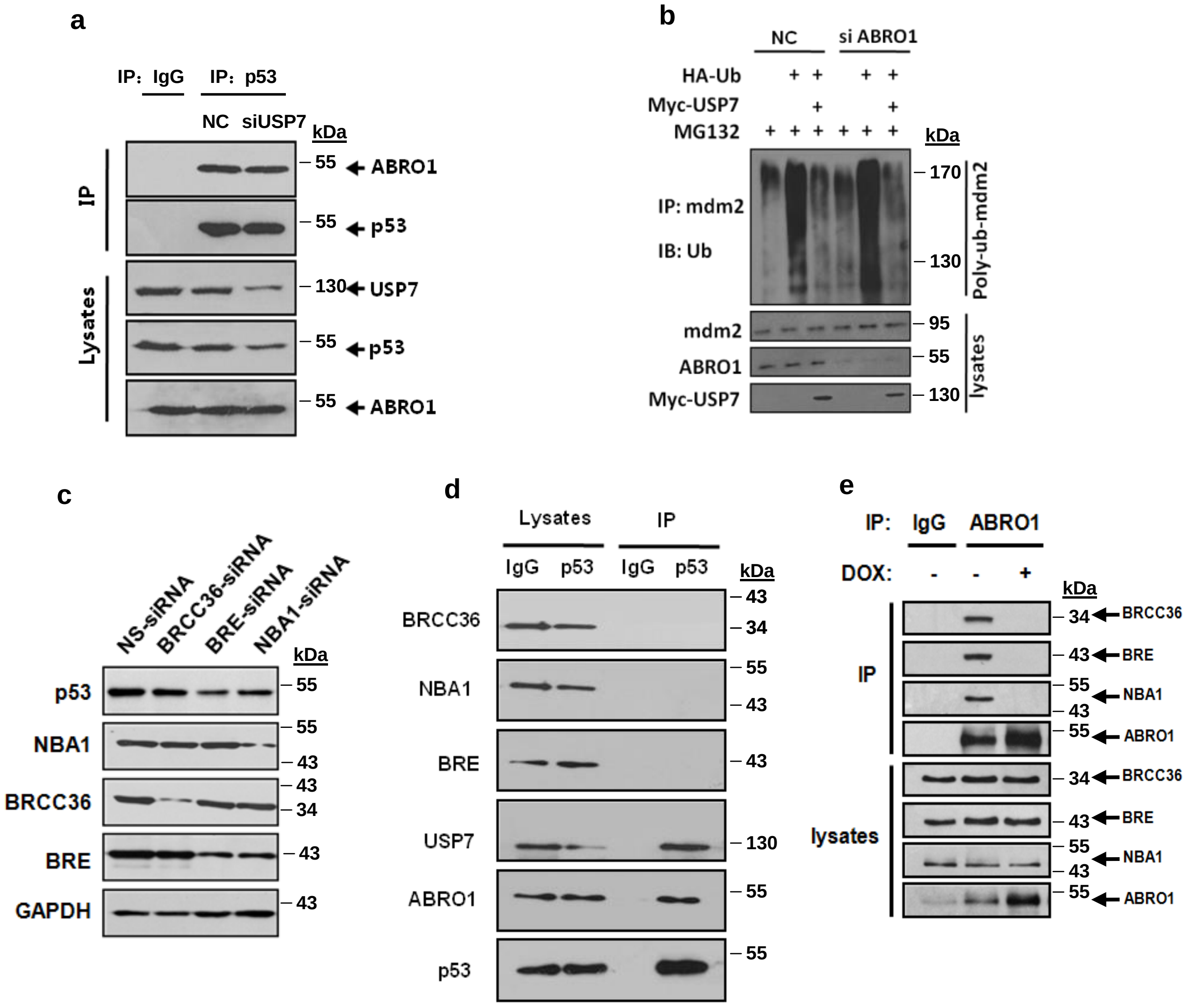

Supplementary Figure 8. ABRO1 regulates p53 stability independent on the BRISC complex. (a) HCT 116 p53$^{+/+}$ cells was transfected with USP7 siRNA or control siRNA, after 48hours, the lysates were immunoprecipitated using p53-specific antibody and analyzed by immunoblotting as indicated. **(b)** HCT116 p53$^{-/-}$cells co-transfected with the indicated constructs and NC-siRNA or ABRO1 siRNA were treated with MG132 for 4 hours before harvest. Lysates were immunoprecipitated with anti-mdm2 antibody and analyzed by immunoblotting

with anti-Ub antibody. **(c)** HCT116 $p53^{+/+}$ cells were transfected with siRNAs as indicated, after 48 hours, the lysates were analyzed by immunoblotting as indicated. **(d)** HCT116 $p53^{+/+}$ cell lysates were immunoprecipitated using IgG or p53-specific antibody and analyzed by immunoblotting as indicated. **(e)** HCT 116 $p53^{+/+}$ cells were treated with or without DOX(2μg/ml), after 12 hours, lysates were immunoprecipitated using *ABRO1*-specific antibody or IgG and analyzed by immunoblotting as indicated.

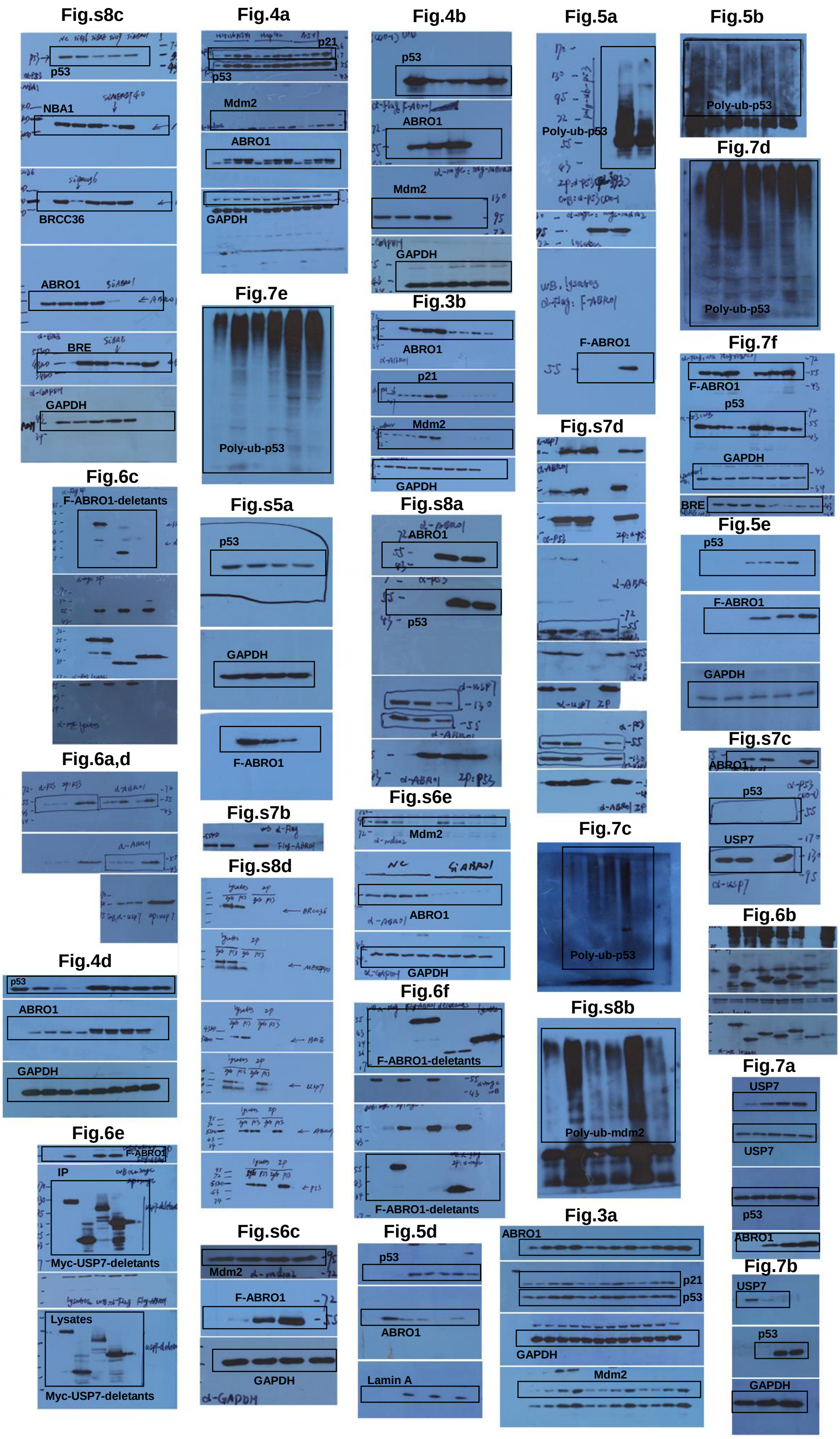

Supplementary Figure 9. Original scanned radiography films of western blots are provided for the corresponding panels.

Supplementary Table 1. Univariate and multivariate analysis of factors associated with survival of HCC patients.

Clinical variables	Patients number	Hazard ratio (95% CI)	p value
Cohort 1			
Univariate analysis[1]			
ABRO1(low versus high)	44/46	0.5(0.3-1.1)	<0.05
Sex (male versus female)	77/13	0.8(0.3-1.9)	0.616
Age (>50 versus ≤50 years)	65/25	1.0(0.5-2.0)	0.971
HBV (positive versus negative)	88/2	0.05(0-550.5)	0.524
Cirrhosis (yes versus no)	23/67	1.0(0.5-2.0)	0.886
Tumour size (≥5 cm versus <5 cm)	39/51	0.5(0.2-0.9)	0.021
TNM stage (Ⅱ/Ⅲ versus Ⅰ)	70/20	0.4(0.2-1.0)	0.051
Multivariate analysis[2]			
ABRO1(low versus high)	44/46	0.5(0.3-1.0)	0.044
Cirrhosis (yes versus no)	23/67	1.1(0.5-2.2)	0.806
Tumour size (≥5 cm versus <5 cm)	39/51	2.0(1.0-4.0)	0.068
TNM stage (Ⅱ/Ⅲ versus Ⅰ)	70/20	1.8(0.6-5.0)	0.287
Cohort 2			
Univariate analysis[1]			
ABRO1(low versus high)	96/65	2.9(1.8-4.6)	<0.001
Sex (male versus female)	139/22	1.0(0.5-1.7)	0.782
Age (>50 versus ≤50 years)	68/93	0.8(0.5-1.2)	0.229
HBV (positive versus negative)	157/4	21(0.2-2763)	0.216
Cirrhosis (yes versus no)	90/71	1.8(1.2-2.8)	0.008
AFP（≥500 versus <500 ng/ml)	78/83	1.2(0.8-1.9)	0.318
Tumour size (≥5 cm versus <5 cm)	101/60	2.8(1.7-4.5)	<0.001
TNM stage (Ⅱ/Ⅲ versus Ⅰ)	102/59	2.8(1.7-4.6)	<0.001
Multivariate analysis[2]			
ABRO1(low versus high)	96/65	0.4(0.2-0.6)	<0.001
Cirrhosis (yes versus no)	90/71	1.8(1.1-2.8)	0.013
Tumour size (≥5 cm versus <5 cm)	101/60	2.1(0.9-5.1)	0.094
TNM stage (Ⅱ/Ⅲ versus Ⅰ)	102/59	1.2(0.5-2.9)	0.693

Analysis was conducted on HCC patients of Cohort 1, Cohort 2 as indicated. Hazard ratios (95% confidence interval) and p values were calculated using univariate or multivariate Cox proportional hazards regression in SPSS 19.0.

[1] The median value in each cohort was chosen for the cut-off point.

[2] Multivariate analysis was adjusted by sex and age.

Abbreviations: CI, confidence interval; HBV, hepatitis B virus; TNM, tumour-node-metastasis; AFP, alpha-fetoprotein.

Supplementary Table 2. Pearson Correlation analysis of each factors associated with survival of HCC patients.

Correlations

		Age	sex	HBV	HCV	cirrhosis	stage	ABRO1	time	status	AFP	size
Age	Pearson Correlation	1	.047	-.025	.a	-.102	-.139	-.012	.188	-.008	-.250	-.190
	Sig. (2-tailed)		.551	.752	.	.200	.079	.884	.017	.919	.001	.016
	N	161	161	161	161	161	161	161	161	161	161	161
sex	Pearson Correlation	.047	1	-.064	.a	.047	.014	.106	-.048	-.007	-.193	.052
	Sig. (2-tailed)	.551		.424	.	.551	.855	.180	.544	.935	.014	.516
	N	161	161	161	161	161	161	161	161	161	161	161
HBV	Pearson Correlation	-.025	-.064	1	.a	-.061	.117	-.194	-.188	-.143	-.005	.117
	Sig. (2-tailed)	.752	.424		.	.439	.138	.014	.017	.071	.950	.138
	N	161	161	161	161	161	161	161	161	161	161	161
HCV	Pearson Correlation	.a	.a	.a	.a	.a	.a	.a	.a	.a	.a	.a
	Sig. (2-tailed)	.	.	.	.	.	.	.	.	.	.	.
	N	161	161	161	161	161	161	161	161	161	161	161
cirrhosis	Pearson Correlation	-.102	.047	-.061	.a	1	.134	.042	-.289	-.091	-.015	.057
	Sig. (2-tailed)	.200	.551	.439	.		.091	.593	.000	.249	.849	.474
	N	161	161	161	161	161	161	161	161	161	161	161
stage	Pearson Correlation	-.139	.014	.117	.a	.134	1	-.196	-.412	-.225	.192	.844
	Sig. (2-tailed)	.079	.855	.138	.	.091		.013	.000	.004	.015	.000
	N	161	161	161	161	161	161	161	161	161	161	161
ABRO1	Pearson Correlation	-.012	.106	-.194	.a	.042	-.196	1	.359	.225	-.012	-.170
	Sig. (2-tailed)	.884	.180	.014	.	.593	.013		.000	.004	.876	.031
	N	161	161	161	161	161	161	161	161	161	161	161
time	Pearson Correlation	.188	-.048	-.188	.a	-.289	-.412	.359	1	.413	-.133	-.386
	Sig. (2-tailed)	.017	.544	.017	.	.000	.000	.000		.000	.092	.000
	N	161	161	161	161	161	161	161	161	161	161	161
status	Pearson Correlation	-.008	-.007	-.143	.a	-.091	-.225	.225	.413	1	-.006	-.225
	Sig. (2-tailed)	.919	.935	.071	.	.249	.004	.004	.000		.940	.004
	N	161	161	161	161	161	161	161	161	161	161	161
AFP	Pearson Correlation	-.250	-.193	-.005	.a	-.015	.192	-.012	-.133	-.006	1	.192
	Sig. (2-tailed)	.001	.014	.950	.	.849	.015	.876	.092	.940		.015
	N	161	161	161	161	161	161	161	161	161	161	161
size	Pearson Correlation	-.190	.052	.117	.a	.057	.844	-.170	-.386	-.225	.192	1
	Sig. (2-tailed)	.016	.516	.138	.	.474	.000	.031	.000	.004	.015	
	N	161	161	161	161	161	161	161	161	161	161	161
